# α-Synuclein-induced dysregulation of neuronal activity contributes to murine dopamine neuron vulnerability

**DOI:** 10.1038/s41531-021-00210-w

**Published:** 2021-08-18

**Authors:** Abeer Dagra, Douglas R. Miller, Min Lin, Adithya Gopinath, Fatemeh Shaerzadeh, Sharonda Harris, Zachary A. Sorrentino, Jonatan Fullerton Støier, Sophia Velasco, Janelle Azar, Adetola R. Alonge, Joseph J. Lebowitz, Brittany Ulm, Mengfei Bu, Carissa A. Hansen, Nikhil Urs, Benoit I. Giasson, Habibeh Khoshbouei

**Affiliations:** 1 Department of Neuroscience, University of Florida, Gainesville, FL USA; 2 Department of Pharmacology and Therapeutics, University of Florida, Gainesville, FL USA; 3 Molecular Neuropharmacology and Genetics Laboratory, Department of Neuroscience, Faculty of Health and Medical Sciences, University of Copenhagen, Copenhagen, Denmark

**Keywords:** Cellular neuroscience, Neurodegeneration, Neurotransmitters

## Abstract

Pathophysiological damages and loss of function of dopamine neurons precede their demise and contribute to the early phases of Parkinson’s disease. The presence of aberrant intracellular pathological inclusions of the protein α-synuclein within ventral midbrain dopaminergic neurons is one of the cardinal features of Parkinson’s disease. We employed molecular biology, electrophysiology, and live-cell imaging to investigate how excessive α-synuclein expression alters multiple characteristics of dopaminergic neuronal dynamics and dopamine transmission in cultured dopamine neurons conditionally expressing GCaMP6f. We found that overexpression of α-synuclein in mouse (male and female) dopaminergic neurons altered neuronal firing properties, calcium dynamics, dopamine release, protein expression, and morphology. Moreover, prolonged exposure to the D2 receptor agonist, quinpirole, rescues many of the alterations induced by α-synuclein overexpression. These studies demonstrate that α-synuclein dysregulation of neuronal activity contributes to the vulnerability of dopaminergic neurons and that modulation of D2 receptor activity can ameliorate the pathophysiology. These findings provide mechanistic insights into the insidious changes in dopaminergic neuronal activity and neuronal loss that characterize Parkinson’s disease progression with significant therapeutic implications.

## Introduction

Volitional movement is a fundamental behavior of everyday life that is often taken for granted until control deteriorates. Dopaminergic neurons within the ventral midbrain play a critical role in the initiation and control of volitional movement^1,2^ and the progressive demise of these neurons is a defining hallmark of Parkinson’s disease (PD)^3^. Pathophysiological damages and loss of function of these neurons precedes their demise and contribute to the early phases of the movement impairments^4^. No current therapies reverse or slow the progression of PD or the many related neurodegenerative diseases associated with the demise of dopaminergic neurons due to the incomplete understanding of the etiology of PD.

The presence of aberrant intracellular inclusions comprised of the protein α-synuclein (α-syn) in the form of Lewy bodies and Lewy neurites within ventral midbrain dopaminergic neurons is another cardinal feature of PD^5–7^. Several missense mutations in the α-syn gene (*SNCA*), as well as duplication^8^ or triplication^9^ of *SNCA*, are sufficient to cause familial PD and the related disease Lewy body dementia. Thus, only a 50% increase in the expression of wild-type α-syn as in the multiplication of the *SNCA* gene is sufficient for a detrimental outcome on dopaminergic neurons resulting in disease. Furthermore, some studies indicate that elevated α-syn level also occurs in idiopathic PD, but the pathophysiological mechanisms associated with increased levels of α-syn remain poorly understood.

The location of midbrain dopamine neurons in deep neural structures creates a significant barrier of not only investigation but also control over the experimental milieu. For these reasons, we incorporated a primary culture model system of dopaminergic neurons, which provides unparalleled access and control over the experimental procedure to investigate potential mechanisms of how excessive α-syn level alters dopaminergic neuronal dynamics and dopamine transmission prior to neuronal demise. Importantly, using whole-cell current-clamp recording, we and others have shown that postnatally derived dopaminergic cultures exhibit spontaneous firing properties^10–14^ similar to those found in ex vivo^15–17^ and in vivo^18–20^. Thereby, the experimental model system used in this study provides mechanistic insights not achievable of those found in ex vivo and in vivo models.

The present study utilized complementary approaches in molecular biology, electrophysiology, and live-cell imaging to investigate the hypothesis that elevated α-syn expression in dopaminergic neurons perturbs intracellular calcium signaling, protein homeostasis, and dopamine transmission prior to neuronal demise. We demonstrated that D2 receptor (D2R) autoinhibition contributes to alterations in neuronal homeostatic properties and that modulation thereof can ameliorate the pathophysiology resulting from excessive α-syn levels. These results provide mechanistic insights into the pathobiological impact of α-syn on dopaminergic neuron function and their demise characteristic of PD.

## Results and discussion

### Tyrosine hydroxylase (TH) promoter-driven adeno-associated virus (AAV) efficiently transduces cultures of midbrain dopamine neurons

**Fig. 1:**
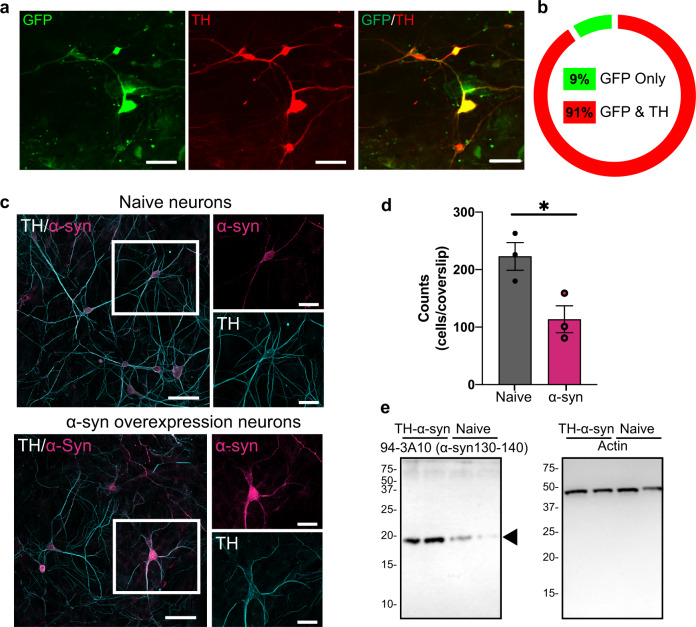
Tyrosine hydroxylase (TH) promoter-driven adeno-associated virus (AAV) efficiently transduces human α-synuclein or the control construct (TH-GFP) in midbrain dopamine neurons. **a**, **b** Immunolabeling of TH confirmed 91 ± 3% of TH-positive neurons co-express GFP, suggesting a high fidelity for pAAV1-TH-GFP viral transduction in the TH-positive neurons (*n* = 3 independent experiments). **c**, **e** The transduction specificity was confirmed via immunocytochemistry analysis and western blot (*n* = 3 independent experiments). Scale bars: 50 μm. **d** Dopaminergic neuron counts revealed that α-syn overexpression decreases neuronal survival (naive = 223 ± 33.52, α-syn = 113.7 ± 33.52, two-tailed unpaired *t* test, naive vs. α-syn, *p* = 0.03), *p < 0.05.

In order to investigate the pathophysiological changes associated with α-syn overexpression, we first developed a cell model with high-fidelity AAV-mediated α-syn expression in midbrain dopaminergic neuronal culture. The ventral midbrain neuronal culture contains the dopaminergic nuclei substantia nigra (SNc) and ventral tegmental area (VTA) that have been frequently used to study dopamine transmission^10–13,21–25^. Notably, SNc dopaminergic neurons are more sensitive than VTA dopaminergic neurons^22,26–31^. Therefore, midbrain neuronal culture is likely to contain more VTA dopaminergic neurons than SNc dopaminergic neurons^22,26–31^ (see limitations of this model system in the “Methods” section). We utilized a TH promoter-driven AAV to specifically express wild-type human α-syn in cultured dopaminergic neurons. First, to demonstrate the specificity of the TH promoter vector, cultures were transduced with AAV-TH-GFP for visual confirmation of expression and quantification of dopaminergic specificity. As demonstrated in Fig. 1a, b, 91 ± 3% of neurons expressing green fluorescent protein (GFP) are TH positive, indicating high specificity. The same pAAV1-TH backbone, but with the human α-syn complementary DNA (cDNA) only, was utilized to overexpress human α-syn in dopamine neurons. The transduction of pAAV1-TH-human-αsyn in cultured midbrain dopamine neurons was confirmed via immunocytochemistry (ICC) and western blot analyses, demonstrating elevated expression of α-syn in these neurons (Fig. 1c, e and Supplementary Fig. 1, *p* = 0.005, two-tailed *t* test, *n* = 3 independent experiments). It should be noted that the midbrain neuronal culture contains many cell types, i.e., neuronal and non-neuronal cells. While our model provided 91% transduction specificity of α-syn overexpression in TH-positive neurons, it is likely that α-syn is also expressed in 9% of TH-negative cells—a mix of neuronal and glial cells—which is a limitation of AAV transduction in general. As shown in Fig. 1d, α-syn overexpression decreases neuronal survival as identified by TH-positive neurons expressing GFP (Fig. 1d, *p* < 0.05). It should be noted that functional analyses are conducted on surviving neurons. Since SNc neurons exhibit increased sensitivity to degeneration, it is possible that the relative proportion of VTA neurons to SNc neurons in cultures is altered by α-syn overexpression. No pre or post hoc techniques were carried out to investigate this ratio or selectively record from a subset of DA cells. Thus, these data may be over-representative of the response of surviving VTA cells; to investigate this question in real-time experiments, future studies with genetic targeting of SNc or VTA subtypes of dopamine neurons will be required (neurod6, pit3x, calbindin, aldh1, and GIRK3).

### Overexpression of α-synuclein disrupts calcium dynamics and firing activity of dopamine neurons

**Fig. 2:**
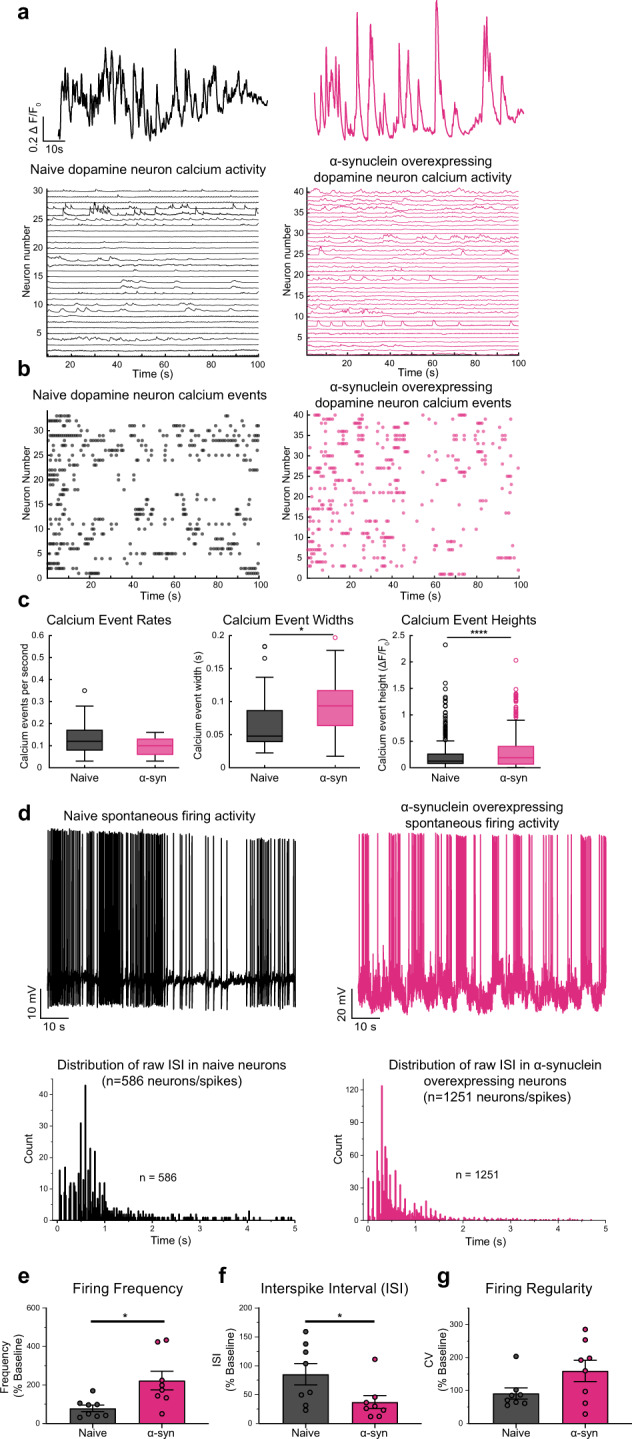
Overexpression of α-synuclein disrupts calcium dynamics and firing activity of dopaminergic neurons. **a** (Top) Representative spontaneous calcium activity in naive dopaminergic neurons (left, black) and dopaminergic neurons overexpressing α-syn (right, pink) exemplify the alteration in calcium dynamics due to increased levels of α-syn. (Bottom) Spontaneous calcium activity encompassing all neurons recorded in each experimental group (*n* = 33 wild-type neurons, *n* = 40 α-syn-overexpressing neurons, form eight biological replicates). **b** Calcium events in all neurons. **c** Spontaneous calcium event rate, width, and amplitude. Overexpression of α-syn does not alter calcium event rate (two-tailed unpaired *t* test, WT vs. α-syn, *p* = 0.1775, *n* = 33 wild-type neurons, *n* = 40 α-syn-overexpressing neurons), α-syn burden broaden calcium events (*p* = 0.0152, two-tailed unpaired *t* test, WT vs. α-syn, *n* = 33 wild-type neurons, *n* = 40 α-syn-overexpressing neurons) and increases in amplitude (*p* = 0.0000198, two-tailed unpaired *t* test, WT vs. α-syn, *n* = 33 wild-type neurons, *n* = 40 α-syn-overexpressing neurons). **d** Representative whole-cell current-clamp recordings of spontaneously active naive (top left, black) compared with overexpressing α-syn dopaminergic neurons (top right, pink). Distribution of raw interspike intervals (ISIs) in naive (bottom left) and α-syn-overexpressing neuron (bottom right) (raw ISI distribution, Kolmogorov–Smirnov test, *D* = 0.26529, *p* < 0.001). **e** Naive compared with α-syn-overexpressing dopaminergic neurons (firing frequency: from eight independent experiments, 100 ± 21.21 for naive neurons vs. 281.7 ± 61.30 for α-syn-overexpressing neurons, two-tailed unpaired *t* test, *p* = 0.0142; **f** interspike interval (ISI): 100 ± 21.63 for naive neurons compared to 43.55 ± 13.28 for α-syn-overexpressing neurons, two-tailed unpaired *t* test, *p* = 0.0431) and **g** firing regularity trend in bursts with intermediated periods of quiescence (CV of ISI—100 ± 18.59 for naive neurons vs. 174.9 ± 35.20 for α-syn-overexpressing neurons, two-tailed unpaired *t* test, *p* = 0.0808). Empty circles in panel **c** represent statistical outliers included in the analyses. Bar graphs ± SEM are overlaid with individually filled data points. *p < 0.05; ****p < 0.0001.

Increased α-syn burden in dopamine neurons is correlated with neuronal loss in neurodegenerative diseases such as PD^32,33^. Although extensively studied in cortical neurons, yeast, and heterologous expression systems^34–39^, α-syn regulation of intracellular calcium and firing activity in dopaminergic neurons prior to cell death remains less clear. The maintenance of calcium homeostasis is a vital process in neurons^40–42^. Calcium is a ubiquitous second messenger that helps to transmit depolarization status and synaptic activity to the biochemical machinery of a neuron^43^. Extensive calcium signaling requires high ATP consumption to restore basal (low) intracellular calcium levels. Increased intracellular calcium may also lead to increased generation of mitochondrial reactive oxygen species^44,45^. Failure to maintain cellular energy levels and to suppress oxygen species may impact calcium signaling during aging and in neurodegeneration^5,6^. To investigate if α-syn overexpression regulates dopaminergic neuronal activity prior to neuronal demise, we employed live-cell calcium imaging in dopamine neurons conditionally expressing GCaMP6f under the control of the dopamine transporter (DAT) promoter (DAT-GCaMP6f) containing either endogenous levels of α-syn (naive) or overexpressing α-syn. Compared to calcium events in naive dopaminergic neurons, both width and amplitude of calcium peaks were increased in the presence of α-syn overexpression, creating repeated burdens on the neuron (Fig. 2a–c, width—*p* = 0.0152, unpaired two-tailed *t* test, *n* = 33 wild-type neurons, *n* = 40 α-syn-overexpressing neurons, amplitude—*p* = 0.0000198, unpaired two-tailed *t* test, *n* = 33 wild-type neurons, *n* = 40 α-syn-overexpressing neurons). These data suggest that increased levels of α-syn in dopamine neurons lead to disturbances in calcium homeostasis that can alter biophysical properties of neurons, neuronal activity, neurotransmission^42,43,46–50^, and neuronal death, all of which are shared hallmarks in neurodegenerative diseases^22,46,51,52^.

While measurement of calcium activity provides inferential information about the neuronal firing, we did not observe a change in calcium event rates (Fig. 2c, left). The firing rate of dopamine neurons ranges from 0.5 to 20 spike/s^10–21^. We and others have shown that electrophysiological properties of cultured dopamine neurons are consistent with those of dopamine neurons in vivo or in vitro^10,11,14,21^. Therefore, next, we investigated whether α-syn overexpression modulates the firing activity of dopaminergic neurons. We utilized whole-cell current-clamp recordings to measure the spontaneous firing activity of cultured DAT-GCaMP6f neurons with endogenous levels or overexpression of α-syn, which were identified by GCAMP6f fluorescence. While dopaminergic neurons containing endogenous levels of α-syn exhibited characteristic pace-making activity^53,54^, the spontaneous firing activity of α-syn-overexpressing dopamine neurons showed an irregular and clustered firing pattern with increased burst firing activity within the clusters (Fig. 2d, e, data are expressed as the percent of control from eight independent experiments, firing frequency—100 ± 21.21 for naive neurons vs. 281.7 ± 61.30 for α-syn-overexpressing neurons, two-tailed unpaired *t* test, naive vs. α-syn, *p* = 0.0142, raw ISI distribution, Kolmogorov–Smirnov test, *D* = 0.26529, *p* < 0.001). Thus far, our data suggest that increased α-syn levels in dopaminergic neurons lead to altered calcium dynamics and increased firing activity. Both firing activity and calcium dynamics in dopamine neurons are tightly regulated by the activity of the D2 autoinhibitory receptors^53–57^. Therefore, we asked whether α-syn-induced dysregulation of dopamine neuronal activity and calcium dynamics is due to reduced D2R autoinhibition.

### α-Syn overexpression reduces D2R-mediated autoinhibition

**Fig. 3:**
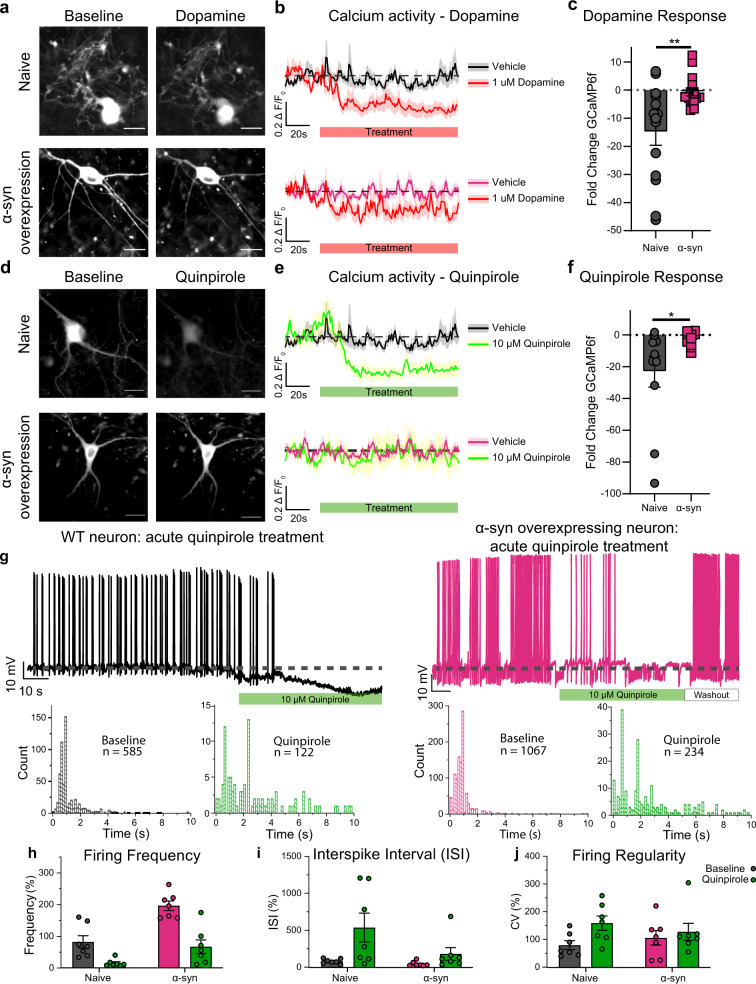
α-Synuclein overexpression reduces D2 receptor autoinhibition. **a** Representative images of naive dopaminergic neurons (top) and α-syn-overexpressing dopaminergic neurons (bottom); before (left) and during dopamine (1 μM) administration (right). **b** (Top) In naive neurons, dopamine reduced the ∆*F*/FGCaMP6f (*n* = 14–21, two-way ANOVA, *p* = 0.0003, from five independent replicates). (Bottom) In α-syn-overexpressing neurons, dopamine produced a smaller reduction in ∆*F*/FGCaMP6f (*n* = 17–26, two-way ANOVA, *p* = 0.0088, from five independent replicates). **c** The fold change in ∆*F*/FGCaMP6f before and after (*n* = 11–17, two-tailed *t* test, *p* = 0.0031, from five independent replicates). **d** Representative images of naive dopaminergic neurons (top) and α-syn-overexpressing neurons (bottom), before (left) and after quinpirole (10 μM) (right). **e** In naive neurons, quinpirole reduced the ∆*F*/FGCaMP6f (top panel) (*n* = 11–21, two-way ANOVA, *p* = 0.0007, from five independent biological replicates). In α-syn-overexpressing neurons, quinpirole produced a smaller reduction in ∆*F*/FGCaMP6f (bottom panel) (*n* = 13–26, two-way ANOVA, *p* = 0.7259, from five independent replicates). **f** Fold change in ∆*F*/FGCaMP6f before and after drug (*n* = 11–13, two-tailed *t* test, *p* = 0.0217, from five independent replicates). **g** (Top left) A representative recording of the spontaneous firing activity of naive dopaminergic neurons before and during quinpirole (10 μM) (*n* = 6, from three independent biological replicates). (Top right) A representative recording of the spontaneous firing activity of α-syn-overexpressing neurons before and during quinpirole (10 μM) (*n* = 8, from three independent experiments). (Bottom) Acute quinpirole treatment significantly increases raw interspike interval distributions of naive and α-syn-overexpressing dopaminergic neurons (Kolmogorov–Smirnov test, naive—*D* = 0.50642, *p* < 0.001, α-syn overexpressing—*D* = 0.47776, *p* < 0.001). **h** Comparison of the firing frequency of naive (black) and α-syn-overexpressing neurons (pink bar) during quinpirole administration (green) (*n* = 7 from independent experiments, two-way ANOVA, *p* = <0.0001). **i** Interspike interval—*n* = 7 from independent experiments, two-way ANOVA, *p* = 0.0136; **j** firing regularity (CV of ISI)—*n* = 7 from independent experiments, two-way ANOVA, *p* = 0.0200). The data are presented as mean ± SEM. **h**, **i** are presented as %change from untreated naive. Scale bar = 50 μm. *p < 0.05, **p < 0.01.

Multiple channels and transporters regulate neuronal activity and intracellular calcium dynamics. Dopamine activation of D2 autoinhibitory receptors on dopamine neurons decreases neuronal activity^16,53,54,56,58^ and intracellular calcium dynamics^52,55,59–62^. Therefore, we tested the hypothesis that the observed disturbance in neuronal excitability and calcium dynamics in α-syn-overexpressing neurons is due to dysregulation of canonical D2R autoinhibition in these neurons. In a double-blinded experimental design, we exposed DAT-GCaMP6f cultures to dopamine (1 µM) while monitoring the change in GCaMP6f signal. Consistent with the literature^63,64^, dopamine induced a decrease in intracellular Ca^2+^ as measured by a decrease in GCaMP6f fluorescence in non-transduced neurons (Fig. 3a–c (top), *n* = 14–21, naive neurons, two-way repeated-measures analysis of variance (ANOVA), row factor: time, % of total variation = 7.65, *p* = 0.0003, column factor: dopamine treated vs. untreated, % of total variation = 6.289, *p* = 0.0003; interaction: time × treatment, % of total variation = 5.079, *p* = <0.0001). However, α-syn-overexpressing neurons exhibited an attenuated dopamine-induced reduction of GCaMP6f signal, although still significantly affected (Fig. 3b, *n* = 17–26, two-way ANOVA where the variables are time and treatment, followed by Tukey’s honestly significant difference (HSD), *p* = 0.0088, from five independent replicates; α-syn-overexpressing neurons, two-way repeated-measures ANOVA, row factor: time, % of total variation = 3.759, *p* = 0.0169, column factor: dopamine treated vs. untreated, % of total variation = 3.189, *p* = 0.0088; interaction: time × treatment, % of total variation = 2.886, *p* = <0.0001). Further, fold-change responses of naive dopamine neurons were greater than α-syn-overexpressing neurons (Fig. 3c, *n* = 11–17, two-tailed unpaired *t* test, naive vs. α-syn, *p* = 0.0031, from five independent replicates; a comparison was made between average calcium activity 30 s before drug and last 30 s of drug exposure). These data suggest that α-syn overexpression decreases the inhibitory feedback regulation in dopamine neurons.

While these data support the interpretation that α-syn overexpression decreases the feedback modulation of neuronal activity, they do not unequivocally show a decrease in D2R activity. Dopamine interacts with multiple targets, such as DAT, that also regulate neuronal excitability^10,11,25,65^ and intracellular calcium activity^10,11,21,23,25^. Therefore, we utilized quinpirole (10 µM), a D2R-specific agonist, to stimulate D2R in DAT-GCaMP6f cultures containing either endogenous levels of α-syn or its overexpression (Fig. 3d). Consistent with the literature^60,62,63,66^, quinpirole activation of D2 autoinhibitory receptors induced the canonical suppression of calcium dynamics in naive dopaminergic neurons, whereas the calcium activity in α-syn-overexpressing neurons did not change during quinpirole administration (Fig. 3e, f naive: *n* = 11–21, two-way repeated-measures ANOVA, row factor: time, % of total variation = 9.923, *p* = <0.0001, column factor: untreated vs. quinpirole, % of total variation = 4.951, *p* = 0.007; interaction: time × pharmacology, % of total variation = 7.233, *p* = <0.0001; α-syn overexpressing: *n* = 11–21, two-way repeated-measures ANOVA, row factor: time, % of total variation = 1.873, *p* = 0.4797, column factor: untreated vs. quinpirole, % of total variation = 0.08018, *p* = 07259; interaction: time × pharmacology, % of total variation = 1.288, *p* = 0.9999; fold-change comparisons—*n* = 11–13, two-tailed unpaired *t* test, naive vs. α-syn, *p* = 0.0217).

Although live-cell calcium imaging provides a proxy for dopaminergic neuronal activity^23^, calcium imaging does not reveal changes in firing activity at the resolution of electrophysiological recordings. Therefore, as a complementary approach, we utilized whole-cell current-clamp recordings to compare the firing activity of naive and α-syn-overexpressing dopaminergic neurons following quinpirole administration. Consistent with the literature, quinpirole activation of D2 autoreceptors decreased the firing activity of naive dopamine neurons^16,56,67–69^ and α-syn-overexpressing dopaminergic neurons (Fig. 3g–j, interspike interval distributions of naive and α-syn-overexpressing dopaminergic neurons (Kolmogorov–Smirnov test, naive—*D* = 0.50642, *p* < 0.001, α-syn overexpressing—*D* = 0.47776, *p* < 0.001), firing frequency—*n* = 7 from independent experiments, 100 ± 23.621 baseline and 18.639 ± 5.300 quinpirole for naive neurons vs. 238.638 ± 18.321 baseline and 81.565 ± 56.046 for α-syn treated with quinpirole, two-way repeated-measures ANOVA, row factor: naive vs. α-syn, % of total variation = 28.69, *p* = 0.0023, column factor: baseline vs. quinpirole, % of total variation = 40.14, *p* = <0.0001; interaction: phenotype × pharmacology, % of total variation = 4.048, *p* = 0.0041; interspike interval—*n* = 7 from independent experiments, 100 ± 13.282 baseline and 782.847 ± 282.0257 quinpirole for naive neurons vs. 51.203 ± 10.741 baseline and 266.5666 ± 124.1509 for α-syn neurons treated with quinpirole, two-way repeated-measures ANOVA, row factor: naive vs. α-syn, % of total variation = 9.071, *p* = 0.1019, column factor: baseline vs. quinpirole, % of total variation = 20.61, *p* = 0.0136; interaction: phenotype × pharmacology, % of total variation = 6.025, *p* = 0.1441; firing regularity (CV of ISI)—*n* = 7 from independent experiments, 100 ± 14.750 baseline and 149.313 ± 24.054 quinpirole for naive neurons vs. 159.960 ± 28.831 baseline vs. 120.241 ± 28.489 for α-syn neurons treated with quinpirole, two-way repeated-measures ANOVA, row factor: naive vs. α-syn, % of total variation = 0.033, *p* = 0.9258, column factor: baseline vs. quinpirole, % of total variation = 13.78, *p* = 0.0200; interaction: phenotype × pharmacology, % of total variation = 4.442, *p* = 0.1540). While acute quinpirole produced the canonical silencing of naive dopaminergic neurons^70,71^ (DAT-Cre-GCaMP6f) (Fig. 3g, left), to our surprise, following acute exposure to quinpirole, the firing activity of α-syn-overexpressing dopamine neurons (DAT-Cre-GCaMP6f) began to resemble baseline firing activity observed in naive dopaminergic neurons (Fig. 3g, left black trace: naive dopamine neuron before and after drug application; Fig. 3g—right pink trace: α-syn neurons before and after drug application) (unpaired two-tailed *t* test, *p* = 0.6096, naive baseline, 0.972 ± 0.230, vs. α-syn in the presence of quinpirole, 0.793 ± 0.253). Collectively, these data support the hypothesis that α-syn overexpression reduces D2R-mediated autoinhibition in cultured dopamine neurons, and suggests that a more prolonged activation of D2R can potentially restore this deficit.

### α-Syn overexpression increases intracellular and extracellular dopamine levels and TH expression

**Fig. 4:**
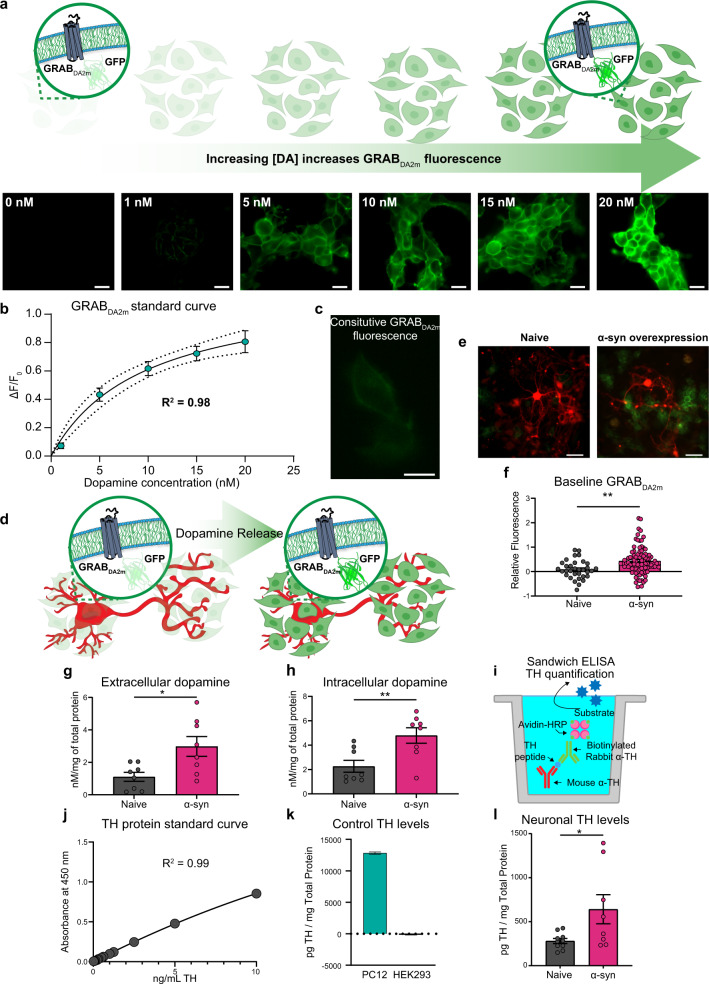
Overexpression of α-synuclein increases intracellular and extracellular dopamine levels with concurrent increased tyrosine hydroxylase expression. **a** Schematic and representative baseline-subtracted images of GRABDA_2M_-HEK cells exposed to increasing concentration of dopamine. Scale bar = 20 μm. **b** A standard curve of GRABDA_2M_-HEK cells against known extracellular dopamine concentrations (*R*^2^ = 0.98). **c** Constitutive GRABDA_2M_-HEK cell fluorescence signal in the absence of dopamine neurons (in culture). Scale bar = 10 μm. **d** Schematic of GRABDA_2M_-HEK cells seeded into dopaminergic cultures. In the presence of dopamine, GRABDA_2M_-HEK cells rapidly increase in fluorescence intensity. **e** Baseline fluorescence levels denote unstimulated and spontaneous dopamine release from the neurons. The average ratio of the fluorescence signal of the cells adjacent to neuron soma and neuronal processes to the average ratio of GRABDA_2M_-HEK cells (only) were calculated (relative fluorescence = (*F*GRABDA2M-HEK cells grown with neurons − *F*_c_)/*F*_c_). GRABDA_2M_-HEK cells cocultured with naive and α-syn-overexpressing neurons. Scale bar = 50 μm. **f** GRABDA_2M_-HEK cells cocultured with α-syn-overexpressing neurons show higher basal fluorescence, indicating higher baseline dopamine release (relative fluorescence) compared to naive neurons (*n* = 10 from three independent replicates; the data are means ± SEM, two-tailed *t* test, *p* = 0.0013). **g**, **h** HPLC analysis complements the GRABDA_2M_-HEK results. Extracellular milieu (**g**) and cell lysate intracellular milieu (**h**) revealed increased intracellular and extracellular dopamine levels in α-syn-overexpressing neurons compared to naive neurons (*n* = 8 each, from eight independent replicates, two-tailed *t* test; intracellular: *p* = 0.0071; extracellular: *p* = 0.0139). **i** Schematic diagram of quantitative ELISA experimental design for TH in dopaminergic neurons. **j** Standard curve for TH sandwich ELISA shows average absorbance values for each purified TH protein concentration from multiple consecutive experiments (*R*^2^ = 0.99). **k** TH protein levels were detected and quantified in positive control groups, PC12 cells, whereas no protein was detected in the negative control group, HEK293 cells. **l** α-Syn-overexpressing neurons exhibited increased levels of TH compared to naive (*n* = 8–10, two-tailed *t* test, *p* = 0.0289). These experiments were performed through a double-blinded experimental design. *p < 0.05, **p < 0.01.

Our findings so far suggest that α-syn may induce a feedforward adaptive mechanism that decreases the ability of inhibitory D2 autoreceptors to act as a brake on neuronal excitability and increase extracellular dopamine levels^53,56^. To test this hypothesis, we used two complementary approaches of high-performance liquid chromatography (HPLC) analysis and an engineered dopamine sensor to measure intracellular and extracellular dopamine levels, *at baseline*. First, we used GRABDA_2M_ (G-protein-coupled receptor activation-based DA-expressing HEK293 cells) to measure extracellular dopamine levels. GRABDA_2M_ is a genetically encoded fluorescent dopamine sensor that is engineered by coupling a conformationally sensitive circular-permutated enhanced GFP (cpEGFP) to D2R. In GRABDA_2M_-expressing HEK293 cells, dopamine binding to the sensor induces a conformational change that results in a robust increase in fluorescence signal in a concentration-dependent manner (Fig. 4a, b).

Constitutive GRABDA_2M_ fluorescence signal in the absence of dopamine neurons was obtained at the beginning of each experiment, where GRABDA_2M_-expressing HEK293 cells were plated in similar conditions, but sans neurons (*F*_c_, Fig. 4c). To compare *baseline* dopamine release amongst the experimental groups, the average ratio of the fluorescence signal of cells adjacent to the soma and neuronal processes to the average ratio of the fluorescence signal of GRABDA_2M_ cells (only) was calculated $\left( {{{{F}}}_{{{{\mathrm{baseline}}}}} = \left( {{{{F}}}_{{{{\mathrm{GRABDA2M}}}}\;{{{\mathrm{cells}}}}\;{{{\mathrm{grown}}}}\;{{{\mathrm{with}}}}\;{{{\mathrm{neurons}}}}}{{{- F}}}_{{{\mathrm{c}}}}} \right)/{{{F}}}_{{{\mathrm{c}}}}} \right)$.

To confirm that the increase in GRABDA_2M_ fluorescence signal is due to dopamine release, we ran a positive control experiment (Supplementary Fig. 2), where GRABDA_2M_ fluorescent signal around the soma and neuronal processes was measured following KCl (90 mM) stimulation of dopamine release^72^. The average fluorescence signal of cells adjacent to the soma and neuronal processes before and after KCl was calculated $\left( {{\Delta}{F}/{F}} = {{(F_{\mathrm{stimulated}}} - {F_{\mathrm{baseline}}})/{F_{\mathrm{baseline}}}} \right)$ (Supplementary Fig. 2A, B). KCl-induced neuronal depolarization^73,74^ produced a robust fluorescence increase in both experimental groups (Supplementary Fig. 2B, *n* = 10 from three independent replicates; the data are mean ± SEM, two-tailed *t* test, *p* = 0.8991). The KCl-evoked dopamine release was similar in both experimental groups.

After confirming the ability of GRABDA_2M_ cells to detect evoked dopamine release, we measured spontaneous (i.e., *baseline, unstimulated*) dopamine release in naive and α-syn-overexpressing neurons. The neurons were cocultured with GRABDA_2M_ cells 20–24 h prior to live-cell confocal imaging. This experimental design enables real-time detection of endogenous dopamine released *at baseline*, i.e., *spontaneous dopamine release* (Fig. 4d, e). Using a blinded experimental design, we found a significantly higher spontaneous dopamine level, as measured by a higher GRABDA_2M_ fluorescence signal around the soma and neuronal processes of α-syn-overexpressing neurons (Fig. 4f, *n* = 33 naive, 89 α-syn-overexpressing from three independent replicates; the data are mean ± SEM, two-tailed *t* test, *p* = 0.0013). These data support the interpretation that α-syn overexpression increases spontaneous neuronal activity, leading to increased extracellular dopamine levels. We previously reported that α-syn overexpression decreases dopamine uptake via the DAT^75^ and we have also reported that α-syn overexpression increases the DAT-mediated dopamine efflux^24^. These data are consistent with the hypothesis that α-syn overexpression can increase extracellular dopamine levels. Collectively, our previous reports^72,73^, combined with the data shown in Figs. 2–4, provide a reasonable cellular mechanism for the puzzling observation by Lam et al.^76^ that in mice overexpressing α-syn there is an initial increase in extracellular dopamine levels in the striatum prior to neuronal death.

Because GRAB_DA_ readouts report only the relative difference in dopamine release between α-syn and control neurons, we used HPLC to measure absolute dopamine levels in the external milieu of α-syn-transduced and naive DAT-GCaMP6f cultures (i.e., spontaneous dopamine release) via a blinded experimental design. HPLC analysis showed significantly higher extracellular dopamine levels in α-syn-overexpressing neurons compared to naive neurons (Fig. 4g, h; *n* = 8 each, from eight independent replicates; two-tailed *t* test, *p* = 0.0139). Collectively, these data, combined with live-cell detection of extracellular dopamine levels (at baseline), support the notion that α-syn modulation of dopaminergic neuronal activity leads to increased extracellular dopamine levels. An increase in extracellular dopamine could be due to increased neuronal activity, increased dopamine synthesis, or both possibilities. Since we have already examined the former (Figs. 2 and 3) to test the latter possibility, we used HPLC to measure intracellular dopamine levels. The measurement of dopamine in the cell lysate of naive and α-syn-overexpressing neurons revealed significantly higher intracellular dopamine levels (Fig. 4h, *n* = 8 each, from eight independent replicates, two-tailed *t* test, *p* = 0.0071). These data suggest that the decreased autoinhibition of dopamine neurons following α-syn overexpression not only increases neuronal excitability but also dysregulates dopamine synthesis and secretion. Furthermore, in Fig. 2, we showed that increased neuronal α-syn increases the magnitude and duration of intracellular calcium burden, which would promote increased basal dopamine release.

Multiple mechanisms likely contribute to the increased intracellular dopamine following α-syn overexpression. For example, increased dopamine uptake via the DAT, decreased DAT-mediated dopamine efflux, increased expression of TH (a key enzyme involved in dopamine synthesis), or a combination of these mechanisms would possibly contribute to a higher intracellular dopamine level. Previously, we and others have shown that α-syn overexpression reduces dopamine recycling by reducing dopamine uptake^75,77,78^. In addition, we have shown that α-syn overexpression increases reverse transport of dopamine, i.e., dopamine efflux^24^, without changing surface DAT levels. Therefore, α-syn regulation of dopamine uptake or dopamine efflux would decrease intracellular dopamine and not increase it.

While α-syn regulation of DAT activity predicts a decrease of intracellular dopamine level^24,75,77–79^, D2R activity negatively regulates TH protein levels as a compensatory mechanism to downregulate dopamine synthesis^56,62,80–83^. As shown in Figs. 2 and 3, we found a reduction in the canonical D2R autoinhibition of dopamine neurons, likely modulating downstream signaling cascades that can regulate TH protein levels. Therefore, next, we tested the hypothesis that in α-syn-overexpressing neurons a decrease in the D2 activity (shown in Figs. 2 and 3) leads to increased TH levels that can contribute to increased intracellular dopamine^80,82–87^. Since the frequently used approaches of western blotting or ICC do not provide purely quantitative data of protein expression to test this hypothesis, we developed an enzyme-linked immunosorbent assay (ELISA)^88^ to quantify TH levels in α-syn-overexpressing neurons (Fig. 4i). For these experiments, we used HEK293 cells as a negative control group and PC12 cells as a positive control group, and a purified full-length recombinant TH protein was used to generate a standard curve (Fig. 4j, k). α-Syn-overexpressing neurons show significantly higher TH levels compared to naive (Fig. 4l, *n* = 8–10, two-tailed *t* test, *p* = 0.0289). While ELISA provides quantitative data for total TH level across these experimental groups, a limitation of this assay is that it cannot discriminate TH phosphorylation that is associated with TH activity and thus dopamine synthesis^80,89,90^. Nevertheless, these data support the interpretation that increased intracellular dopamine in α-syn-overexpressing neurons, at least in part, is due to increased TH protein levels.

### Altered neural dynamics mediated by α-syn may emerge from altered D2 activity and expression patterns

**Fig. 5:**
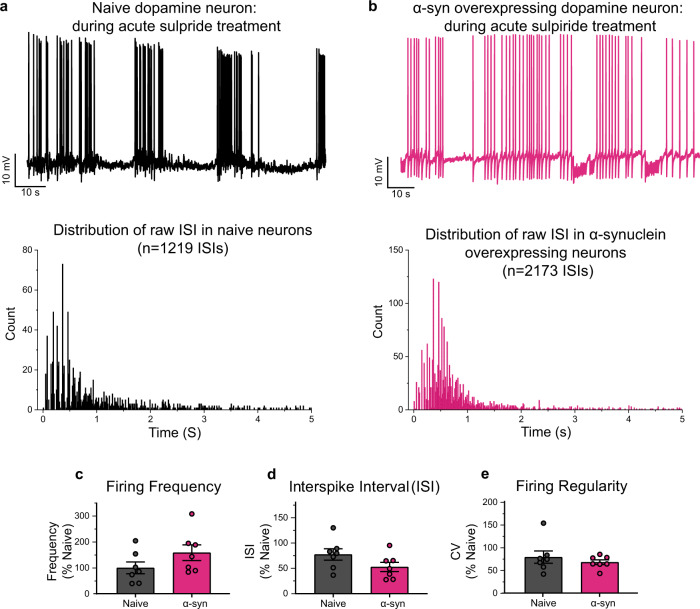
D2 receptor antagonism in dopaminergic neurons mimics burst firing pattern with a significantly higher firing frequency observed in α-synuclein-overexpressing dopamine neurons that presents with lower membrane/cytoplasmic D2 ratio. **a**, **b** Representative whole-cell current-clamp recordings of spontaneously active naive (**a**, top, black) and α-syn-overexpressing (**b**, top pink) dopaminergic neurons during sulpiride (D2 antagonist, 5 μM) bath application. **a**, **b** (Bottom) Distribution of raw ISIs in naive (**a**, bottom) and α-syn-overexpressing (**b**, bottom) dopaminergic neurons (Kolmogorov–Smirnov test, *D* = 0.13114, *p* < 0.001). **c**–**e** The bar graph shows firing frequency (**c**), interspike interval (ISI) (**d**), and firing regularity (**e**) during bath application of sulpiride (5 μM), revealing D2 antagonism in naive dopaminergic neurons promotes firing rates, interspike intervals, and regularity comparable to neurons overexpressing α-syn (*n* = 8 from three independent biological replicates, two-tailed unpaired *t* test, firing frequency: 100 ± 22.94 naive vs. 158.8 ± 30.37 α-syn-overexpressing neurons, *p* = 0.148; ISI: 100 ± 14.51 naive vs. 68.15 ± 11.84 α-syn-overexpressing neurons, *p* = 0.1147; CV of ISI: 100 ± 17.02 naive vs. 85.94 ± 6.599 α-syn-overexpressing neurons, *p* = 0.456).

Our data, thus far, support the interpretation that the canonical D2R-mediated autoinhibition, such as inhibitory modulation of spontaneous firing activity, is reduced in α-syn-overexpressing neurons. While D2R agonist quinpirole silenced naive dopamine neurons, the response to quinpirole in α-syn-overexpressing neurons is significantly reduced, possibly due to desensitization or reduced activity of the D2R (Fig. 3). Therefore, next, we tested the hypothesis that blockade of D2Rs in naive dopaminergic neurons simulates the firing activity observed in α-syn-overexpressing neurons. We performed whole-cell current-clamp recordings to measure spontaneous firing activity of dopaminergic neurons before and during bath application of sulpiride (D2 antagonist, 5 µM). In naive dopaminergic neurons, bath application of sulpiride produced burst firing patterns with intermediated periods of quiescence and firing frequencies similar to α-syn-overexpressing dopaminergic neurons in the presence of sulpiride (Fig. 5a–e, *n* = 8 from three independent biological replicates, distribution of raw ISIs in naive and α-syn-overexpressing dopaminergic neurons, Kolmogorov–Smirnov test, *D* = 0.13114, *p* < 0.001, two-tailed unpaired *t* test, firing frequency: 100 ± 22.94 naive vs. 158.8 ± 30.37 α-syn-overexpressing neurons, *p* = 0.148; ISI: 100 ± 14.51 naive vs. 68.15 ± 11.84 α-syn-overexpressing neurons, *p* = 0.1147; CV of ISI: 100 ± 17.02 naive vs. 85.94 ± 6.599 α-syn-overexpressing neurons, *p* = 0.456). These data support the hypothesis that α-syn-overexpressing dopamine neurons exhibit reduced functional availability of D2-mediated response, which could be due to receptor desensitization^89,90^, decreased membrane expression of D2Rs, or a combination of these possibilities.

To investigate if α-syn overexpression in dopaminergic neurons alters D2R expression, we performed cell surface biotinylation of D2R via a blinded experimental design with striatal lysate as a positive control group and CHO cells as the negative control group. When total D2R was normalized to HSP60 loading control, there were no significant differences between naive and α-syn-overexpressing neurons (Supplementary Fig. 3, from three independent biological replicates, two-tailed unpaired *t* test, *p* = 0.3417). We also found that the ratio of cytoplasmic D2R to total D2R in α-syn-overexpressing neurons was not significantly different from naive neurons (Supplementary Fig. 3, from three independent biological replicates, two-tailed unpaired *t* test, *p* = 0.9426). However, when comparing the ratio of membrane-to-cytoplasmic D2R, we found that α-syn-overexpressing neurons have a significantly lower ratio of membrane-to-cytoplasmic D2R than naive neurons (Supplementary Fig. 3B, from three independent biological replicates, two-tailed unpaired *t* test, *p* = 0.0039). It should be noted that biotinylation assay detects total (both functional and desensitized receptors). The double ICC of fixed, but not permeabilized, dopamine neurons stained for both D2R and an integral membrane protein such as Na^+^/K^+^-ATPase, or GM1-CTxB would have been a suitable complementary approach to examine membrane-localized D2R across the experimental groups in this study. However, the frequently used D2R antibodies in the field^91–94^ are raised against the intracellular N-terminal domain of the receptor. This limitation decreases the confidence in the identification of membrane vs. intracellular protein levels. A similar technical limitation applies to the single-cell qPCR assay, where total transcript levels do not necessarily reflect functional D2Rs at the membrane. The latter limitation somewhat applies to the biotinylation assay used in this study. Unless an antibody is raised against the active or inactive form of the receptor, a biotinylation assay detects both functional and desensitized receptors. Therefore, although our data suggest that membrane D2Rs are decreased in α-syn-overexpressing neurons, it is possible that the detected membrane D2Rs are desensitized, i.e., a lesser receptor–effector coupling^95–98^. Therefore, live-cell functional assays, such as electrophysiology and calcium imaging, combined with pharmacological manipulations are more reliable strategies to assess the mechanism of α-syn regulation of neuronal activity.

### α-Syn overexpression reduces arborization of dopamine neurons and pretreatment with a D2R agonist partially rescues the detrimental impact of α-syn

Dopaminergic neurons have extensive axonal arborizations and large terminal fields^99–101^, where one dopamine neuron is estimated to have ~245,000 release sites^102,103^. Studies in animal models of PD and postmortem data in human PD^104^ show that decreased axonal complexity and dendritic arborization, reduction of the number of axon terminals, and global neuronal size precede neuronal death^77,102,105^. Our data suggest that prior to cell death, via a D2R mechanism, α-syn overexpression can induce neuronal disinhibition, leading to increased intracellular and extracellular dopamine levels that are implicated in increased neuronal vulnerability^35,106,107^. Therefore, we investigated the potential link between α-syn-mediated dopamine neuronal dysfunction and neuronal complexity.

**Fig. 6:**
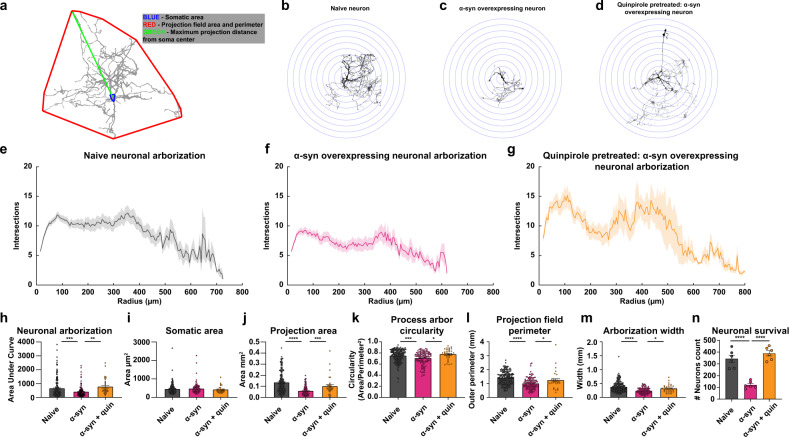
α-Synuclein overexpression reduces arborization of dopaminergic neurons and treatment with a D2 receptor agonist partially rescues the detrimental impact of α-synuclein. Experiments conducted in at least three independent biological replicates. **a** Schematic representation of morphological analysis. **b**–**d** Representative binarized images of naive (**b**), α-syn-overexpressing (**c**), and quinpirole-pretreated α-syn-overexpressing neurons (**d**). Sholl intersection profiles of untreated naive (**e**), untreated α-syn-overexpressing (**f**), and quinpirole-pretreated α-syn-overexpressing neuron (**g**) measurement of area under curve (**h**) (one-way ANOVA, naive *n* = 190, α-syn, *n* = 114, naive vs. α-syn, *p* = 0.0009). Sholl analyses revealed that 48 h quinpirole (0.5 μM) pretreatment partially restores arborization complexity compared to untreated α-syn-overexpressing neurons (one-way ANOVA, naive *n* = 190, α-syn *n* = 114, and α-syn + quinpirole *n* = 32, naive vs. α-syn *p* = 0.0009, naive vs. α-syn + quinpirole *p* = 0.5131, and α-syn vs. α-syn + quinpirole *p* = 0.0041). **i** Somatic areas were comparable between experimental groups (one-way ANOVA, naive *n* = 190, α-syn *n* = 114, naive vs. α-syn *p* = 0.67). **j** α-Syn-overexpressing neurons project over smaller area than naive neurons (one-way ANOVA, naive *n* = 190, α-syn *n* = 114, naive vs. α-syn *p* = 0.0001). **k**–**m** Detrimental morphological changes in α-syn-overexpressing neurons (one-way ANOVA, naive *n* = 190, α-syn *n* = 114, circularity: naive vs. α-syn *p* = 0.0021, outer perimeter: naive vs. α-syn *p* = 0.0001; width: naive vs. α-syn *p* = 0.0001). D2 receptor agonist partially rescues the detrimental impact of α-synuclein. Quinpirole treatment of α-syn-overexpressing neurons rescued changes in (**j**) projection area (one-way ANOVA, naive *n* = 190, α-syn *n* = 114, and α-syn + quinpirole *n* = 32, naive vs. α-syn *p* = 0.0001, naive vs. α-syn + quinpirole *p* = 0.1136, and α-syn vs. α-syn treated with quinpirole *p* = 0.0486), (**k**) neuronal circularity, (**l**) projection field perimeter, and (**m**) arborization width (one-way ANOVA, naive *n* = 190, α-syn *n* = 114, and α-syn + quinpirole *n* = 32, circularity: naive vs. α-syn *p* = 0.0021, naive vs. α-syn + quinpirole *p* = 0.4452, and α-syn vs. α-syn + quinpirole *p* = 0.0046; outer perimeter: naive vs. α-syn *p* = 0.0001, naive vs. α-syn + quinpirole *p* = 0.2250, and α-syn vs. α-syn + quinpirole *p* = 0.0312; width: naive vs. α-syn *p* = 0.0001, naive vs. α-syn + quinpirole *p* = 0.1419, and α-syn vs. α-syn + quinpirole *p* = 0.0187). **n** Dopaminergic neuron counts revealed that α-syn overexpression decreases neuronal survival, which is rescued when pretreated with quinpirole (0.5 μM for 48 h) (one-way ANOVA, naive vs. α-syn *p* = 0.0008, naive vs. α-syn + quinpirole *p* = 0.1364, and α-syn vs. α-syn + quinpirole *p* = 0.0021). *p < 0.05, **p < 0.01, ***p < 0.001, ****p < 0.0001.

Sholl analysis entails using concentric circles around the soma of a neuron, with neurite fields intersecting these concentric circles counted as a measure of differences in neuronal complexity (Fig. 6a–d). This analytical approach estimates^99,106–108^ neuronal complexity via assessment of projection area, number of intersections as a measure of neuronal arborization, projection field perimeter, neuronal arborization width, and circularity of neurite arborization^108–113^. Compared to naive dopaminergic neurons, α-syn-overexpressing neurons exhibit a lower degree of neurite arborization (Fig. 6e, f, h), reduced projection area (Fig. 6j), reduced process arbor circularity (Fig. 6k), smaller projection field perimeter (Fig. 6l), and smaller arborization width (Fig. 6m), but no change in the soma area (Fig. 6i, one-way ANOVA followed by Tukey’s HSD, naive *n* = 190, α-syn *n* = 114, intersections: naive vs. α-syn, *p* = 0.0009, circularity: naive vs. α-syn, *p* = 0.0021, outer perimeter: naive vs. α-syn, *p* = 0.0001; width: naive vs. α-syn, *p* = 0.0001, projection area: naive vs. α-syn, *p* = 0.0001, soma area: naive vs. α-syn, *p* = 0.67, from at least three independent biological replicates). The loss of neuronal complexity and decreased dendritic arborization found in this study are consistent with morphological data in postmortem PD samples^104^, potentially informing the progression of α-syn-induced pathology prior to neuronal loss.

The unexpected observation that acute D2 treatment, as shown in Fig. 3g (right), restored firing properties of α-syn-overexpressing neurons to the levels observed in naive dopamine neurons, supports the hypothesis that pharmacological activation of D2R could be a potential target to alleviate α-syn pathology prior to neuronal death. This hypothesis is consistent with reports showing that D2R activation sustains the structural plasticity of dopaminergic neurons by maintaining their dendritic arborization^107,114–117^. It has been reported that PD is proceeded by retrograde axonal degeneration^118,119^ and D2Rs’ activity regulates dopaminergic neuronal complexity^107,114–117^. Therefore, based on the literature and our data in Fig. 3g, next we tested the hypothesis that prolonged D2R activation would ameliorate the reduction of neuronal complexity^107,120,121^ in the presence of α-syn burdens. We measured neuronal complexity when α-syn-overexpressing neurons were pretreated with quinpirole (0.5 µM) for 48 h. Surprisingly, we found that quinpirole produced a marked improvement in arborization of α-syn-overexpressing neurons (Fig. 6d, g). Detailed morphometric analysis revealed no change in somatic size of neurons across all experimental groups, whereas quinpirole restored arbor circularity, projection area, projection field perimeter, and width of α-syn-overexpressing dopamine neurons, to the level measured in naive untreated neurons shown in Fig. 6b–m (naive *n* = 190, α-syn *n* = 114 and α-syn + quinpirole *n* = 32, from at least three independent replicates; circularity: naive vs. α-syn *p* = 0.0021, naive vs. α-syn + quinpirole *p* = 0.4452, and α-syn vs. α-syn + quinpirole *p* = 0.0046; outer perimeter: naive vs. α-syn *p* = 0.0001, naive vs. α-syn + quinpirole *p* = 0.2250, and α-syn vs. α-syn + quinpirole *p* = 0.0312; shape factor: naive vs. α-syn *p* = 0.0021, naive vs. α-syn + quinpirole *p* = 0.5574, and α-syn vs. α-syn + quinpirole *p* = 0.0086; width: naive vs. α-syn *p* = 0.0001, naive vs. α-syn + quinpirole *p* = 0.1419, and α-syn vs. α-syn + quinpirole *p* = 0.0187, one-way ANOVA followed by Tukey’s HSD).

Next, we examined the impact of α-syn overexpression on neuronal survival by counting TH-positive neurons via ICC. Compared to naive neurons, we found significantly fewer TH-positive neurons following α-syn overexpression; quinpirole-mediated activation of D2Rs (0.5 µM for 48 h) prevented the neuronal loss (Fig. 6n: naive vs. α-syn *p* = 0.0008, naive vs. α-syn + quinpirole *p* = 0.1364, and α-syn vs. α-syn + quinpirole *p* = 0.0021, from six independent biological replicates, one-way ANOVA followed by Tukey’s HSD). The increased neuronal survival following quinpirole pretreatment is consistent with previous reports^107,114–117^ and supports the interpretation that there is a correlation between α-syn modulation of neuronal complexity, neuronal vulnerability, and neuronal loss^122–124^. Neuronal survival is not equivalent to neuronal viability. While these data suggest that pretreatment with a D2R agonist increases neuronal survival following α-syn overexpression, they do not demonstrate a restoration of neuronal activity. Therefore, next, we asked whether quinpirole pretreatment prevents the increase in action potential (AP) frequency, increased intrav and extracellular dopamine levels, and elevated intraneuronal calcium dynamics in α-syn-overexpressing neurons.

### D2R activation partially restores neuronal activity in α-syn-overexpressing dopamine neurons

**Fig. 7:**
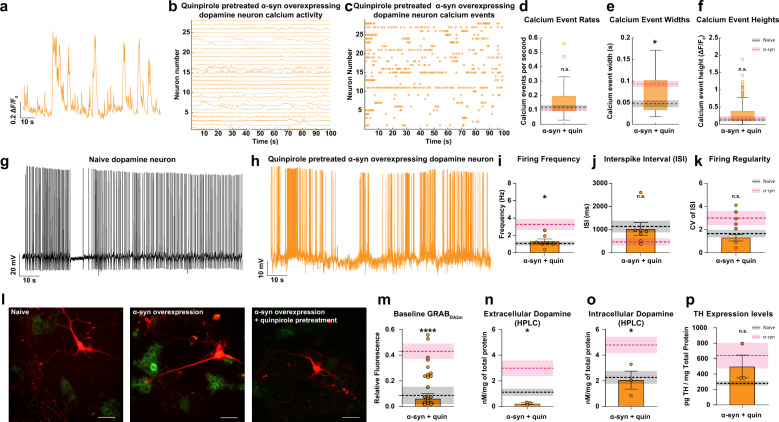
Pretreatment with D2 receptor stimulation partially restores neuronal activity in α-synuclein-overexpressing dopamine neurons. Previous data are overlaid with the dotted line representing averages for untreated naive neurons (black) and untreated α-syn-overexpressing neurons (pink). Shading indicates SEM. **a**, **b** Representative ∆*F*/FGCaMP6f trace (**a**), calcium activity (**b**), and event (**c**) of α-syn-overexpressing neuron pretreated with quinpirole (0.5 μM, 48 h) exhibiting calcium dynamics similar to untreated naive neurons. **d**–**f** Event rate, width, and amplitude after quinpirole pretreatment, respectively (*n* = 28 quinpirole-treated α-syn-overexpressing neurons, two-tailed unpaired *t* test, α-syn vs. α-syn + quinpirole *p* = 0.2024 event rate, *p* = 0.0277 event widths, *p* = 0.6204 event height, untreated α-syn data presented in Fig. 2). Box plot whiskers represent the 95% confidence interval, the upper and lower bounds of the box represent the 75th and 25th percentiles, respectively; the middle line indicates the median value of the sample. Representative firing activity of an untreated naive (**g**) and quinpirole-pretreated α-syn-overexpressing neuron (**h**). **i**–**k** Firing frequency (**i**), interspike interval (**j**), and firing regularity (**k**) in quinpirole-pretreated α-syn-overexpressing neuron (*n* = 7, 1.325 ± 0.2735 Hz for quinpirole-treated α-syn-overexpressing neurons, two-tailed unpaired *t* test, α-syn vs. α-syn + quinpirole *p* = 0.0342 for firing frequency, *p* = 0.1053 for ISI, *p* = 0.4778 for CV of ISI, untreated α-syn data are presented in Fig. 2). **l** GRABDA_2M_-HEKs seeded with untreated naive (left), untreated α-syn-overexpressing (middle), and quinpirole-pretreated α-syn-overexpressing neurons (right). **m** Quinpirole rescued extracellular dopamine level in α-syn-overexpressing neurons (*n* = 6, one-way ANOVA, naive vs. α-syn + quinpirole *p* = 0.9948, α-syn vs. α-syn + quinpirole *p* = 0.0003, untreated α-syn and naive data presented in Fig. 4). **n**, **o** HPLC quantification of dopamine confirm that quinpirole pretreatment of α-syn-overexpressing neurons reduces extracellular (**n**) and intracellular (**o**) dopamine levels vs. untreated α-syn-overexpressing neurons (*n* = 3 each, one-way ANOVA, intracellular: α-syn vs. α-syn + quinpirole *p* = 0.0325 and naive vs. α-syn + quinpirole *p* = 0.9959; extracellular: α-syn vs. α-syn + quinpirole *p* = 0.0449 and naive vs. α-syn + quinpirole *p* = 0.6197, untreated α-syn and naive data presented in Fig. 4). **p** Quantitative TH ELISA (*n* = 3, one-way ANOVA, naive vs. α-syn + quinpirole *p* = 0.4288 and α-syn vs. α-syn + quinpirole *p* = 0.6809, untreated α-syn and naive data are presented in Fig. 4). Data are presented as mean ± SEM, from at least three independent biological replicates. n.s. not significant. *p < 0.05, ****p < 0.0001.

The energy homeostasis principle suggests that the balance between energy income, expenditure, and availability is the key parameter in determining neuronal endurance^125^. APs impose the highest energy demands on neurons^102,125,126^. In addition, dopamine metabolism is strongly linked to oxidative stress, as its degradation generates reactive oxygen species^35,127,128^ that have shown to increase the vulnerability of dopamine neurons to oxidative stress^101,128–134^. So far, we have identified multiple interrelated mechanisms that can potentially lead to the vulnerability of α-syn-overexpressing dopamine neurons. We identified an increase in AP frequency, increased intracellular and extracellular dopamine levels, and elevated intraneuronal calcium dynamics in α-syn-overexpressing neurons that are directly or indirectly related to decreased D2R activity. The unexpected observation that protracted (48 h) application of D2R agonist increased neuronal survival and nearly restored neuronal complexity of α-syn-overexpressing neurons to the levels measured in naive dopaminergic neurons at baseline suggests that the pharmacological activation of D2Rs might be a possible target to alleviate the untoward consequences of α-syn overexpression on neuronal activity prior to neuronal death. To test this hypothesis, we treated α-syn-overexpressing neurons with 0.5 µM quinpirole for 48 h before assessing calcium dynamics, spontaneous firing activity, and dopamine release and synthesis in these neurons (Fig. 7). We compared the results of these experiments to our previous data obtained in α-syn-overexpressing neurons. To reduce the impact of type 1 statistical errors, naive primary midbrain cultures were produced alongside each experimental group.

While it may be intriguing to study the effects of pretreatment of quinpirole on naive neurons, we found that this was not feasible for spontaneous calcium activity and firing as these neurons were silent in the recording chamber (zero values for firing activity that cannot be included in statistical analyses of the data in Fig. 7). Furthermore, D2 autoreceptor activity has been shown to be a potent regulator of neuronal activity^13,58,67,91,135–138^, intracellular calcium^23,50,91,138–140^, neuronal morphology^100,141^, and protein expression^55,90,106,138,140,142–145^. Specifically, the purpose of quinpirole pretreatment was to examine whether modulation of D2R activity would restore or augment the properties of neurons with increased α-syn burdens to those of naive neurons, which has previously been shown to be true for MPP + exposure^117^, and not whether quinpirole pretreatment alters naive neurons. The black dotted line represents the average values measured for untreated naive neurons. The pink dotted line represents the average values for untreated α-syn-overexpressing neurons. The shaded region indicates the respective SEM for each measurement. We found that a prolonged D2R activation partially restores calcium dynamics in these neurons, approximating calcium dynamics measured in untreated naive neurons (Fig. 7a–f, *n* = 28 quinpirole-treated α-syn-overexpressing neurons, two-tailed unpaired *t* test, α-syn vs. α-syn pretreated with quinpirole *p* = 0.2024 event rate, *p* = 0.0277 event widths, *p* = 0.6204 event height, untreated α-syn, and naive data are presented in Fig. 2), suggesting a restoration of calcium homeostasis in these neurons that might be causal or a consequence of a shift in neuronal activity. To test this hypothesis, we employed whole-cell current-clamp recordings to measure the spontaneous firing activity of α-syn-overexpressing neurons after treatment with quinpirole (0.5 µM for 48 h). Quinpirole pretreatment on α-syn-overexpressing neurons decreased the burst firing frequency, shortened the intermediate periods of quiescence, and restored firing regularity near to the values measured in untreated naive neurons (Fig. 7g–k, *n* = 7 from three independent biological replicates, 117.1 ± 21.17 for quinpirole-treated α-syn-overexpressing neurons, two-tailed unpaired *t* test, α-syn vs. α-syn pretreated with quinpirole *p* = 0.0342 for firing frequency, *p* = 0.1053 for ISI, *p* = 0.4778 for CV of ISI, untreated α-syn, and naive data are presented in Fig. 2). These results suggest that dysregulation of D2R in α-syn-overexpressing dopamine neurons can be partially rescued with prolonged activation of the remaining functional D2Rs on the cell surface.

The observed changes in neuronal responses and calcium activity following extended D2R activation could be predictive of downstream changes in dopamine synthesis in α-syn-overexpressing neurons. To test the hypothesis that D2R activation decreases α-syn modulation of dopamine release, we measured extracellular dopamine levels via two complementary approaches: live-cell imaging utilizing an engineered dopamine sensor and HPLC. To measure D2R-mediated modulation of baseline extracellular dopamine levels (0.5 µM quinpirole 48 h) in α-syn-overexpressing neurons, we cocultured GRABDA_2M_-expressing cells with the quinpirole-treated, α-syn-overexpressing dopamine neurons for 20–24 h prior to imaging. Quinpirole pretreatment of α-syn-overexpressing neurons decreased basal GRABDA_2M_ fluorescence (used as a proxy to measure basal dopamine release) around the soma and dendritic fields (Fig. 7l, m), comparable to values measured in naive untreated neurons shown in Fig. 4 (represented as a black dotted line in Fig. 7).

As a complementary approach, we used HPLC, as described in the “Methods” section and in Fig. 4, to measure extracellular dopamine level in the external milieu of neurons after quinpirole pretreatment (0.5 µM for 48 h) via a blinded experimental design. HPLC analysis revealed a reduction in basal dopamine release in all quinpirole-treated experimental groups, with the largest fold decrease in α-syn-overexpressing neurons (Fig. 7n, *n* = 3 from independent biological replicates, one-way ANOVA followed by Tukey’s HSD, α-syn vs. α-syn treated with quinpirole *p* = 0.0325 and naive vs. α-syn treated with quinpirole *p* = 0.9959). Therefore, through pharmacological manipulation of D2Rs, the α-syn dysregulation of dopamine transmission is potentially reversible (*n* = 3 each, from three independent biological replicates, one-way ANOVA followed by Tukey’s HSD, α-syn vs. α-syn treated with quinpirole *p* = 0.0325 and naive vs. α-syn treated with quinpirole *p* = 0.9959). The restoration of extracellular dopamine could be due to decreased neuronal activity, decreased dopamine synthesis, or both. Since we have already examined the former (Fig. 7a–k), to test the possibility of decreased dopamine synthesis, we used HPLC to measure intracellular dopamine levels via a blinded experimental design (described in the “Methods” section). Intracellular dopamine levels in quinpirole-treated α-syn-overexpressing neurons were significantly reduced compared to untreated α-syn-overexpressing neurons (Fig. 7o, *n* = 3 independent biological replicates, one-way ANOVA followed by Tukey’s HSD, α-syn vs. α-syn treated with quinpirole *p* = 0.0449 and naive vs. α-syn treated with quinpirole *p* = 0.6197) shown in Fig. 4 (*n* = 3 each, from 3 independent replicates, one-way ANOVA followed by Tukey’s HSD, α-syn vs. α-syn treated with quinpirole *p* = 0.0325 and naive vs. α-syn treated with quinpirole *p* = 0.9959). Since activation of D2R negatively regulates TH^55,56,80,89,90,146–148^ and neuronal activity^16,53–56,58^, we then tested the hypothesis that reduced intracellular and extracellular dopamine are, in part, due to decreased TH protein levels. Via a blinded experimental design, we utilized quantitative ELISA, as described in Fig. 4, to measure TH levels. As shown in Fig. 7p, TH protein level is similar in quinpirole-treated, α-syn-overexpressing neurons compared to untreated (0.5 µM for 48 h; *n* = 3, one-way ANOVA followed by Tukey’s HSD, naive vs. α-syn treated with quinpirole *p* = 0.4288 and α-syn vs. α-syn treated with quinpirole *p* = 0.6809). The partial rescue of α-syn-induced neuronal dysregulation after D2R activation is consistent with neuroprotective properties of D2Rs described previously^117,149–152^. It has been shown that D2 autoreceptors suppress dopamine synthesis through a negative feedback mechanism, and thus reduce oxidative stress caused by a high level of cytoplasmic dopamine^149–151^. In addition, consistent with our data, activation of D2 autoreceptors mediates neuroprotection by reducing neuronal excitability, cytoplasmic dopamine, and calcium levels^117,152^ that can restore the balance between energy income, expenditure, and its availability^125^. The data presented in this study provide a potential druggable target that may revert or prevent the untoward consequences of α-syn burden on dopamine neuronal activity and viability.

**Fig. 8:**
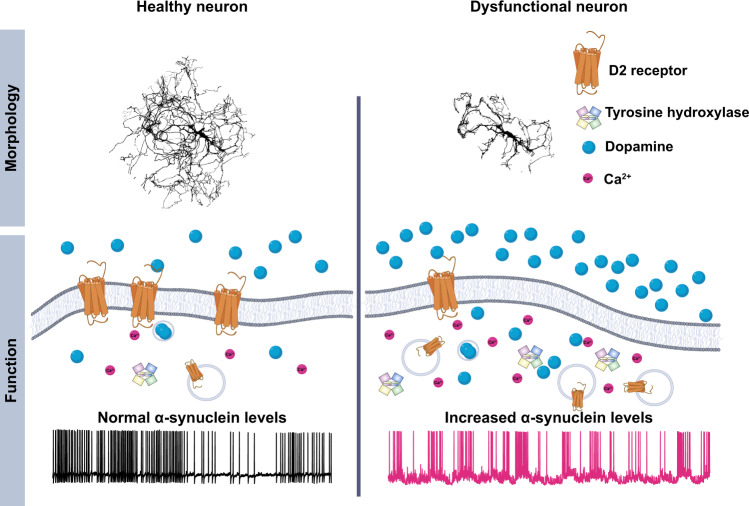
Graphical summary. α-Syn-mediated pathophysiological damages and loss of function of dopamine neurons precede neuronal demise.

To summarize (Fig. 8), we found that α-syn overexpression dysregulates the structural and functional properties of dopaminergic neurons. The untoward consequences of increased α-syn likely cascade across the neuron, protracting the neuronal processes, increasing calcium burdens, and biophysical properties of dopamine neurons as measured by increased burst firing activity. We found that the endogenous self-regulation of dopaminergic neurons fails to restrain the exacerbation of these phenotypes. Thereby, the signaling of these neurons in their networks becomes erratic, potentially creating avalanching neuronal dysfunction. The dysregulation of dopamine signaling within the brain therefore precedes neuronal demise. However, we show that these progressive dysregulations can be reversed through pharmacological manipulation.

The D2 autoreceptor-mediated feedback mechanism is one of the main autoinhibitory mechanisms regulating dopamine neuronal activity^16,56,153^. We found that D2 autoreceptor activity is diminished in α-syn-overexpressing dopamine neurons, and prolonged incubation with a D2R agonist, quinpirole (48 h, 0.5 μM), nearly restored the firing activity to its canonical levels, reinstated intracellular and extracellular dopamine levels, and prevented neuronal loss and structural neural complexity. Notably, D2R agonists (full and partial) have attained Food and Drug Administration approval and have made their way into the clinic; however, these are often tested in late-stage PD. Our results suggest that the current treatment timeline may occur too late and that the efficacy of this strategy requires early intervention to reduce the rate of neuronal demise. Most crucially, our results suggest that neuronal loss might be preventable, and future exploration across other mechanistic pathways will reveal intersectional treatments that may have the capacity to ameliorate PD.

## Methods

When possible, the experiments are performed via a blinded experimental design. Animals were maintained in the University of Florida animal facilities. Experiments were approved by the Institutional Animal Care and Use Committee at the University of Florida.

### Reagents and chemicals

The source, catalog number, and concentration of reagents, antibodies, and chemicals used in this study are outlined in Table 1. All viral vectors utilized in this study are listed in Table 2.

**Table 1:** Biochemical assays.

Dissociation media composition					
Chemical name	Concentration	Vendor	Catalog number		
NaCl	116 mM	Sigma-Aldrich	S7653		
NaHCO_3_	26 mM	Sigma-Aldrich	S5761		
NaH_2_PO_4_	2 mM	Sigma-Aldrich	S9638		
d-glucose	25 mM	Sigma-Aldrich	G8769		
MgSO_4_	1 mM	Sigma-Aldrich	M7506		
Cysteine	1.3 mM	Sigma-Aldrich	C7352		
Papain	400 U/ml	Worthington Biochemical Corporation	LS003127		
Kynurenic acid	0.5 mM	Sigma-Aldrich	K3375		

Glia media composition					
Chemical name	Concentration	Vendor	Catalog number		
DMEM	51.45%	Thermo Fisher Scientific	11330032		
Fetal bovine serum	39.6%	Gemini	100–106		
Penicillin/streptomycin	1%	Thermo Fisher Scientific	15-140-122		
Glutamax 100X	1%	Thermo Fisher Scientific	35050061		
Insulin (25 mg/ml stock)	0.08%	Sigma-Aldrich	I5500		

DIV0 neuronal media composition					
Chemical name	Concentration	Vendor	Catalog number		
Neurobasal-A	96.9%	Thermo Fisher Scientific	10888022		
B27 Plus	1.9%	Thermo Fisher Scientific	A3582801		
GDNF	0.15%	Sigma-Aldrich	SRP3200		
Glutamax 100X	1%	Thermo Fisher Scientific	35050061		
Kynurenic acid	0.08%	Sigma-Aldrich	K3375		

Maintenance of neuronal media composition					
Chemical name	Concentration	Vendor	Catalog number		
Neurobasal-A	97.1%	Thermo Fisher Scientific	10888022		
B27 Plus	1.9%	Thermo Fisher Scientific	A3582801		
Glutamax 100X	0.97%	Thermo Fisher Scientific	35050061		

Immunocytochemistry					
General reagents					
Chemicals	Concentration	Vendor	Catalog number		
PBS	1X	Prepared as needed on-site	N/A		
Triton X-100	0.50%	Thermo Fisher Scientific	bp151-100		
NGS	10%	Lampire Biological Products	7332500		
Paraformaldehyde	4%	Electron Microscopy Sciences	157-4-100		

Primary antibodies					
Primary antibody–antigen	Host species/isotype	Concentration	Vendor	Catalog number	Use
Tyrosine hydroxylase	Rabbit/polyclonal	1:500	EMD Millipore	AB152	ICC/WB
Alpha-synuclein (syn211)	Mouse/IgG1	1:500	Abcam	AB80627	ICC
DAT	Rat	1:500	Millipore Sigma	AB5802	
Calbindin	Rabbit/polyclonal	1:500	EMD Millipore	ABN2192	
GFP	Rabbit/IgG	1:500	Thermo Fischer Scientific	A-11122	ICC
Human α-syn (C-terminal regions, 130–140) (94-3A10)	Mouse/monoclonal IgG1	1:500	Gifted from Dr. Giasson	N/A	WB
Actin C4	Mouse/monoclonal	1:500	EMD Millipore	MAB1501	WB
D2	Mouse	1:500	Neuromab	75–230	WB/ICC
D2	Rabbit	1:500	Millipore	AB5084P	WB

Secondary antibodies					
Conjugate	Host species/target species	Concentration	Vendor	Catalog number	Use
Alexa Fluor 568	Goat/mouse	1:500	Life Technologies	A-21124	ICC
Alexa Fluor 647	Goat/mouse	1:500	Life Technologies	A21242	ICC
Alexa Fluor 488	Goat/rabbit	1:500	Life Technologies	A-11006	ICC
HRP	Goat/mouse	1:2000	Jackson Immuno Research Labs	AB-10015289	WB
HRP	Goat/rabbit	1:2000	Jackson Immuno Research Labs	AB_2307391	WB
IRDye® 800CW	Goat/mouse	1:15000	LI-COR	926-32210	WB

Biotinylation, cell lysis, and ELISA					
ELISA coating buffer, pH 9.6					
Chemical name	Concentration	Vendor	Catalog number		
Na_2_CO_3_	28.3 mM	Sigma-Aldrich	D6546		
NaHCO_3_	71.42 mM	Sigma-Aldrich	S5761		

BufferD lysis buffer, pH 7.6					
Chemical name	Concentration	Vendor	Catalog number		
Glycerol	10% (v/v)	Sigma-Aldrich	G5516		
NaCl	125 mM	Sigma-Aldrich	S7653		
EDTA	1 mM	Sigma-Aldrich	E9884		
EGTA	1 mM	Sigma-Aldrich	3777		

Biotinylation (buffer pH 7.8)					
Chemical name	Concentration	Vendor	Catalog number		
NaCl	150 mM	Sigma-Aldrich	S7653		
CaCl_2_	2 mM	Sigma-Aldrich	449709		
Triethanolamine	10 mM	Sigma-Aldrich	90279		
Sulfo-NHS-SS biotin	1.5 mg/ml	Thermo Fisher Pierce	22331		
Monomeric avidin resin	N/A	Thermo Fisher Pierce	53146		

Antibodies for ELISA					
Specificity	Clone/species	Conjugate	Dilution	Vendor	Catalog number
TH	Monoclonal/mouse	N/A	1:1000	EnCor	MCA-4H2
TH	Polyclonal/rabbit	Biotin	1:1000	EnCor	RPCA-TH
Biotin	N/A (avidin)	HRP	1:2500	Vector Labs	A-2004

Calcium imaging ACSF composition					
pH range: 7.35–7.4; osmolarity range: 305–310					
Chemical name	Concentration	Vendor	Catalog number		
NaCl	126 mM	Sigma-Aldrich	S7653		
KCl	2.5 mM	Sigma-Aldrich	P9541		
CaCl_2_	2 mM	Sigma-Aldrich	223506		
NaH_2_PO_4_	1.25 mM	Sigma-Aldrich	71505		
MgSO_4_	2 mM	Sigma-Aldrich	M7506		
Dextrose	10 mM	Sigma-Aldrich	D9434		
NaHCO_3_	24 mM	Sigma-Aldrich	S5761		

Pharmacological agents					
Resource/reagent	Concentration	Vendor	Catalog number		
Dopamine hydrochloride	1 μM	Sigma-Aldrich	H8502		
Quinpirole hydrochloride	10 μM	Sigma-Aldrich	Q102		

Electrophysiology ACSF composition					
pH range: 7.35–7.4; osmolarity range: 305–310					
Chemical name	Concentration	Vendor	Catalog number		
NaCl	126 mM	Sigma-Aldrich	S7653		
KCl	2.5 mM	Sigma-Aldrich	P9541		
CaCl_2_	2 mM	Sigma-Aldrich	223506		
NaH_2_PO_4_	1.25 mM	Sigma-Aldrich	71505		
MgSO_4_	2 mM	Sigma-Aldrich	M7506		
Dextrose	10 mM	Sigma-Aldrich	D9434		
NaHCO_3_	26 mM	Sigma-Aldrich	S5761		
Potassium gluconate	120 mM	Sigma-Aldrich	P1847		
MgCl_2_	2 mM	Sigma-Aldrich	M8266		
HEPES	10 mM	Sigma-Aldrich	H3375		
EGTA	0.1 mM	Sigma-Aldrich	E3889		
ATPNa_2_	2 mM	Sigma-Aldrich	A2383		
GTPNa	0.25 mM	Sigma-Aldrich	A2383		

Reagents for ELISA					
Reagent	Concentration	Supplier	Catalog number	Purpose	
Fat-free milk	1% or 5%>	Carnation	N/A	WB/ELISA	
TMB substrate	Stock	Thermo Fisher	34028	ELISA	
H_2_SO_4_	2 N	Sigma	339741	ELISA	
Tween-20	0.002	Thermo Fisher	MP1Tween201	TBS-T	
Protease inhibitor	1×	Milipore	539191	Cell lysis	
DC protein assay	N/A	Biorad	5000112	Protein assay	
Immulon 4 HBX	N/A	Thermo Fisher	3855	ELISA	

1× KH buffer (cell lysate)					
pH: 7.4, adjusted with HCl or NaOH as needed					
Chemical name	Concentration	Vendor	Catalog number		
NaCl	118 mM	Sigma-Aldrich	S7653		
KCl	4.7 mM	Sigma-Aldrich	P9541		
CaCl_2_	1.25 mM	Sigma-Aldrich	223506		
KH_2_PO_4_	1.2 mM	Sigma-Aldrich	P5655		
MgSO_4_	1.2 mM	Sigma-Aldrich	M7506		
d-glucose	11 mM	Sigma-Aldrich	G8769		
l-Ascorbic acid	0.5 mg/mL	Sigma-Aldrich	A5960		
NaHCO_3_	25 mM	Sigma-Aldrich	S5761		

HEPES ACSF					
pH: 7.4, adjusted with HCl or NaOH as needed					
Chemical name	Concentration	Vendor	Catalog number		
NaCl	92 mM	Sigma-Aldrich	S7653		
KCl	2.5 mM	Sigma-Aldrich	P9541		
CaCl_2_	0.5 mM	Sigma-Aldrich	223506		
NaH_2_PO_4_	1.2 mM	Sigma-Aldrich	P5655		
NaHCO_3_	30 mM	Sigma-Aldrich	S5761		
MgSO_4_	10 mM	Sigma-Aldrich	M7506		
d-glucose	25 mM	Sigma-Aldrich	G8769		
HEPES	20 mM	Sigma-Aldrich	H3375		
Na-l-ascorbate	5 mM	Sigma-Aldrich	A4034		
Na-pyruvate	3 mM	Sigma-Aldrich	P5280		
Thiourea	2 mM	Sigma-Aldrich	T7875		

*PBS* phosphate-buffered saline, *DAT* dopamine transporter, *GFP* green fluorescent protein, *NGS* normal goat serum, *N/A* not available.

**Table 2:** Virus.

Viral vectors (AAV)	Concentration	Vendor	Catalog number	Capsid serotype	Other
TH-α-syn	3.26.E^7^ U/μl	Dr. Giasson	N/A	AAV1	
TH-GFP	3.26.E^7^ U/μl	Dr. Giasson	N/A	AAV1	
LoxP-Tdtomato	3.26.E^7^ U/μl	Addgene	28306	AAV1	

*N/A* not available.

### Animals

All experiments were approved by the Institutional Animal Care and Use Committee at the University of Florida. Mice were housed in the animal care facility at the University of Florida, 2–4 per cage with food and water available ad libitum in the home cage. The room was maintained under 12 h light/dark cycle. Wild-type C57BL/6J mice, or DAT^IRES*cre*^ and Ai95(RCL-GCaMP6f)-D (Ai95D) knock-in mice were obtained from The Jackson Laboratory (stock number: 006660 (DAT^IRES*cre*^), 024105 (Ai95D), Bar Harbor, ME, USA). C57BL/6J pups or pups expressing GCaMP6f in dopamine neurons were used for this study. Mice of both sexes were used.

### AAV1-TH-α-syn and AAV1-TH-GFP generation

The pAAV2.5-THP-GFP plasmid was purchased from Addgene (#80336)^154^. Human α-syn cDNA from a pcDNA3.1 plasmid was restriction digested with *Eco*RI and *Hin*dIII (NEB), purified, and ligated in the same sites of the pAAV2.5-THP backbone to generate the pAAV2.5-TH-α-syn plasmid. Purified pAAV2.5-TH-α-syn and pAAV2.5-TH-GFP vectors were utilized to prepare active AAV (capsid 1) using a HEK293T-based transfection method followed by iodixanol gradient purification as previously described^155^ and these viruses were termed AAV1-TH-α-syn and AAV1-TH-GFP. The genomic titer of each virus was assayed by quantitative PCR as previously described^155^.

### Primary neuronal culture

Primary culture was prepared as previously described, with small distinctions^23^. Briefly, acutely dissociated mouse midbrains from 0- to 2-day-old male and female pups were isolated and incubated in a dissociation medium at 35–37 °C under continuous oxygenation for 60–90 min. Dissociated cells were triturated with pipettes of decreasing bore size (including a punctured fire-polished pipette), then pelleted by centrifugation at 1500 r.p.m. for 3–5 min, and finally resuspended and plated in glial medium (Table 1). Cells were plated at a density of 100,000 cells/coverslip on a 12 mm coverslip coated with 0.1 mg/ml poly-d-lysine and 5 μg/ml laminin and maintained in neuronal media. After 2 h, cells were supplemented with neuronal media (days in vitro 0 (DIV0) composition). Every 4 days, 1/3 of the media was replaced with fresh media. On DIV5, cultures were transduced with the desired AAV1 (see Table 2). The experiments described in this study were performed on DIV9–11. Reagents and chemicals utilized for midbrain neuronal culture are listed in Table 1.

### Electrophysiology

Spontaneous firing activity of midbrain dopamine neurons was examined via whole-cell current-clamp recordings as previously described^10,11,21^. The neurons were continuously perfused with artificial cerebral spinal fluid (ACSF) (composition is described in Table 1) equilibrated with 95% O_2_/5% CO_2_; pH was adjusted to 7.4 at 37 °C. Patch electrodes were fabricated from borosilicate glass (Cat. No. 1B150F-4, 1.5 mm outer diameter; World Precision Instruments, Sarasota, FL) with the P-2000 puller (Sutter Instruments, Novato, CA). The tip resistance was in the range 3–5 MΩ. The electrodes were filled with a pipette solution containing (in mM): 120 potassium gluconate, 20 KCl, 2 MgCl_2_, 10 HEPES, 0.1 EGTA, 2 ATP, and 0.25 GTP, with pH adjusted to 7.25 with KOH. All experiments were performed at 37 °C. To standardize AP recordings, neurons were held at their resting membrane potential (see below) by DC application through the recording electrode. AP was recorded if the following criteria were met: a resting membrane potential more polarized than −35 mV and an AP peak amplitude >60 mV. AP half-width was measured as the spike width at the half-maximal voltage using Clampfit 10 software (Molecular Devices LLC, San Jose, CA). The steady-state basal activity was recorded for 2–3 min before bath application of the drug. Each coverslip was used for only one recording; this is specifically important for experiments involving drug application. The spontaneous spike activity of midbrain dopamine neurons was obtained by averaging 1 min interval activities at baseline and after 3–5 min of drug exposure.

### Live-cell calcium imaging

Live-cell calcium imaging and analysis are described previously^23^. Briefly, naive (non-transduced) and α-syn-overexpressing (transduced with AAV1-TH-α-syn) midbrain neuronal cultures were imaged with a Nikon Eclipse FN1 upright microscope (Nikon Instruments, Melville, NY). A Spectra X (Lumencor, Inc., Beaverton, OR) was used to stimulate GCaMP6f (*λ*_ex_ = 470 nm) fluorescence through a custom quad-pass filter (Chroma Technologies, Battleboro, VT), and emission was filtered through visible spectra bandpass filter. Experiments were performed under gravity perfusion of ACSF (Table 1). The average fluorescence of the first 60 s recording is defined as the baseline. After baseline imaging, vehicle (ACSF), 1 μM DA, or 10 μM quinpirole was administered via a perfusion system (flow rate of 2 ml/min) and recorded for an additional 2 min. Background fluorescence was subtracted from each frame. Fold fluorescence change from baseline was calculated and plotted against time. Each coverslip was used for only one recording; this is specifically important for experiments involving drug application. All resources and reagents used for live-cell calcium imaging experiments are listed in Table 1.

### Live-cell confocal imaging using GRABDA_2M_-expressing HEK293 cells to measure *extracellular dopamine*

These experiments were performed via a blinded experimental design. GRABDA_2M_ is a genetically encoded fluorescent dopamine sensor that is engineered by coupling a conformationally sensitive cpEGFP to D2R. In GRABDA_2M_-expressing HEK293 cells, dopamine binding to the sensor induces a conformational change that results in a robust increase in fluorescence signal in a concentration-dependent manner^156^. Flp-In™ 293 T-Rex stable cell lines exhibiting tetracycline-inducible expression of the GRABDA_2M_ dopamine sensor were generously gifted by Dr. Ulrik Gether. The GRABDA_2M_-expressing HEK293 cells were maintained in Dulbecco’s modified Eagle’s medium supplemented with 10% fetal bovine serum and 100 U/ml penicillin/streptomycin. Selection pressure for GRABDA_2M_-expressing cells was maintained with media containing Hygro-B (1 mg/ml) and blasticidin (0.015 mg/ml). The cells were plated on coverslips in media containing tetracycline (1:1000) to induce the expression for 12–24 h before live-cell imaging under three conditions: (1) only GRABDA_2M_-expressing HEK293 cells to measure the constitutive fluorescent signal, (2) GRABDA_2M_ cells added to tdTomato-expressing dopaminergic neurons, (3) GRABDA_2M_ cells added to tdTomato-expressing dopaminergic neurons overexpressing α-syn. The tdTomato transduction (*λ*_ex_ = 560 nm) is used for better identification of soma and dendritic field and no fluorophore overlap (i.e., excitation and emission of tdTomato does not bleed through GFP channel). Imaging was performed using a Nikon Eclipse FN1 upright microscope (Nikon Instruments, Melville, NY). A Spectra X (Lumencor, Inc., Beaverton, OR) was used to stimulate GFP (*λ*_ex_ = 470 nm) and tdTomato (*λ*_ex_ = 560 nm) fluorescence through a custom quad-pass filter (Chroma Technologies, Battleboro, VT). Emission was filtered through visible spectra bandpass filter. Regions of interest (ROI) were autodetected via NIS Elements software (Nikon Instruments, Melville, NY). cpEGFP signal was background subtracted using an ROI in an adjacent area of the image devoid of cells or debris.

### Generation of a standard curve

To generate a standard curve, the baseline fluorescence signal (*F*_c_), which is the constitutive fluorescent signal in the absence of extracellular dopamine, was recorded. Changes in fluorescence signal after adding various dopamine concentrations (1–20 nM) were plotted against dopamine concentration.

### Measurement of basal dopamine release

GRABDA_2M_ cells are cocultured with tdTomato-expressing dopamine neurons (DAT^IRES*cre*^-LoxP-tdTomato) containing endogenous α-syn or its overexpression 20–24 h prior to live-cell confocal imaging.

At the beginning of each experiment, the constitutive GRABDA_2M_ fluorescence signal (*F*_c_) of the cells that are plated in similar conditions sans neurons was obtained. To compare baseline dopamine release amongst the experimental groups, the average fluorescence signal of cells adjacent to the soma and neuronal processes to the average fluorescence signal of GRABDA_2M_ cells (only) were calculated in Eq. (1):

1 $$F_{\mathrm{baseline}} = \frac{{F_{{\mathrm{GRAB}}_{\mathrm{DA4.4}}} - F_{\mathrm{c}}}}{{F_{\mathrm{c}}}}$$

### Visualization and quantification of real-time dopamine release following KCl stimulation

Twenty to twenty-four hours prior to live-cell confocal imaging, GRABDA_2M_ cells are cocultured with tdTomato-expressing dopamine neurons containing endogenous α-syn or its overexpression. GRABDA_2M_ fluorescent signal around the soma and neuronal processes were measured before (*F*_baseline_) and following KCl stimulation (90 mM) of dopamine release^69^. The average fluorescence signal of cells adjacent to the soma and neuronal processes before and after KCl were calculated in Eq. (2):

2 $$\frac{{{\Delta}{F}}}{F} = \frac{{F_{\mathrm{stimulated}} - F_{\mathrm{baseline}}}}{{F_{\mathrm{baseline}}}}$$

### Immunocytochemistry

These experiments were performed via a blinded experimental design. On DIV9–11, naive (non-transduced) and α-syn-overexpressing neuronal cultures were fixed with 4% paraformaldehyde (PFA) in PBS for 30 min at room temperature (RT), followed by blocking, permeabilizations, and overnight incubation (at 4 °C) with primary antibodies diluted in blocking buffer, followed by three 20 min phosphate-buffered solution (PBS) washes. Then, a 1 h incubation in blocking buffer with Alexa Fluor-conjugated secondary antibodies at RT, followed by three 20 min washes and an overnight PBS wash at RT. Coverslips were mounted on slides using Fluoromount-G. Images were captured on a Nikon A1 laser-scanning confocal microscope (×20 or ×40 oil-immersion objective). Reagents and chemicals utilized for ICC are listed in Table 1.

### Western blot analysis

These experiments were performed via a blinded experimental design. For detection of endogenous and human α-syn, total cell lysates of neurons transduced with AAV1-TH-α-syn (*n* = 4) or naive (non-transduced) neurons (*n* = 4) were used, as described previously^157^. Briefly, the cells were harvested in 200 µl of 2% sodium dodecyl sulfate (SDS) buffer, protein concentrations were determined using the bicinchoninic acid assay (Pierce), and further diluted in sample buffer (10 mM Tris, pH 6.8, 1 mM EDTA, 40 mM dithiothreitol, 0.005% bromophenol blue, 0.0025% pyronin yellow, 1% SDS, 10% sucrose). Following harvest of total cell lysate, samples were heated to 100 °C for 10 min prior to SDS-polyacrylamide gel electrophoresis (SDS-PAGE) (13% polyacrylamide gels, 10 µg lysate per well) followed by electrophoretic transfer onto 0.2 µm nitrocellulose membranes as previously described^145^. Membranes were incubated with block solution (5% milk in TBS) for 1 h, and then with primary antibodies (in block solution) overnight at 4 °C. Membranes were washed with TBS-T and incubated with goat anti-mouse or anti-rabbit secondary antibodies conjugated to horseradish peroxidase (Jackson Immuno Research Labs, Westgrove, PA) diluted in block solution at RT for 1 h. Immunoreactivity was assessed using Western Lightning-Plus ECL reagents (PerkinElmer, Waltham, MA) followed by chemiluminescence imaging (Genegnome XRQ, Syngene, Frederick, MD). For α-syn detection, we used 94-3A10 antibody, which is a mouse monoclonal antibody raised against C-terminal regions (130–140) of α-syn^158^. For loading control, we used mouse monoclonal anti-actin C4 antibody (EMD Millipore). For TH detection, an affinity-purified rabbit antibody AB152 (EMD Millipore) was used.

### Biotinylation assay

These experiments were performed via a blinded experimental design. α-Syn-overexpressing neuronal cultures and naive neuronal cultures were washed three times with cold PBS and incubated with sulfo-NHS-biotin (1.5 mg/ml; Thermo Fisher Pierce, 21331) for 30 min at 4 °C while rocking. The remaining sulfo-NHS-biotin was quenched with cold Quenching Solution (glycine 50 mM in PBS), followed by three washes with cold PBS^139,159^. Cells were lysed in BufferD lysis buffer (10% glycerol, 125 mM NaCl, 1 mM EDTA, 1 mM EGTA, pH 7.6) containing 1% Triton X-100 and protease inhibitor cocktail (Millipore, 539131) for 1 h at 4 °C while rocking, followed by centrifugation for 15 min at 12,000 × *g*. The supernatants were divided into three portions—25 μl for protein quantification and 200 μl for incubation with avidin, with the remainder for the whole lysate. After equilibrating monomeric UltraLink Avidin (Thermo Fisher Pierce, 53146) twice with 1 ml BufferD, 40 μl of 50% bead slurry were added to 200 μl lysate and incubated at 4 °C for 1 h while rotating. The supernatant was retained as a cytoplasmic fraction, and beads were washed three times with 1 ml BufferD, eluted with 40 μl Laemmli Sample Buffer 4× (containing 10% beta-mercaptoethanol) at 37 °C for 30 min, and separated by 10% SDS-PAGE, transferred to 0.45 μm nitrocellulose, and probed with antibodies against proteins of interest (see Table 1). Fluorescent images were analyzed using ImageJ (NIH) to measure band optical density. Values were normalized to total protein per lane. Beta-tubulin (Aves, TUJ) was probed to demonstrate membrane fraction isolation during biotinylation.

### ELISA quantification of TH

These experiments were performed via a blinded experimental design.

### Cell lysis

For total protein quantification via ELISA, neuronal cultures were washed three times with cold PBS, then lysed in BufferD lysis buffer (10% glycerol, 125 mM NaCl, 1 mM EDTA, 1 mM EGTA, pH 7.6) containing 1% Triton X-100 and protease inhibitor cocktail (Millipore, 539131) for 1 h at 4 °C with rocking, followed by centrifugation for 15 min at 12,000 × *g*. Samples were denatured in Laemmli Sample Buffer 4× (containing 10% beta-mercaptoethanol) at 37 °C for 30 min and separated by 10% SDS-PAGE, transferred to 0.45 μm nitrocellulose, and probed with antibodies against proteins of interest. Values were normalized to total protein per lane.

### TH ELISA

Antibodies and concentrations used are given in Table 1. In brief, Immulon 4 HBX High-Binding 96-well plates were coated with 100 μl per well of 1:1000 dilution of mouse anti-TH (EnCor, MCA-4H2) in coating buffer (28.3 mM Na_2_CO_3_, 71.42 mM NaHCO_3_, pH 9.6) for 20 h at 4 °C. Edge lanes 1 and 12 were left empty. Wells were blocked with 5% fat-free milk in 1× TBS (pH 7.4) for 1 h at RT on an orbital shaker set to 90 r.p.m.

### Generation of a standard curve

To produce a standard curve, two standard curve lanes were generated, with six serial dilutions, beginning at 10 ng/ml and 1 ng/ml in TBS-T containing 1% fat-free milk (with the last well in each standard curve lane left with incubation buffer only as a blank). The remaining wells were incubated in duplicate with lysates from cells of interest. Incubation was completed for 20 h at 4 °C on an ELISA shaker set to 475 r.p.m. After each well was washed and aspirated six times with TBS-T, anti-TH rabbit (EnCor, RPCA-TH) conjugated to biotin was diluted 1:6000 from a stock concentration of 1.65 mg/ml in TBS-T with 1% fat-free milk and incubated for 1 h at RT by centrifugation at 425 r.p.m. One hundred microliters of Avidin-HRP (Vector labs, A-2004), diluted 1:2500 in TBS-T with 1% fat-free milk, was added to each well following washing, and then incubated for 1 h at RT by centrifugation at 425 r.p.m. Following final washes, 150 μl TMB-ELISA reagent (Thermo Fisher, 34028) was added to each well at RT. The reaction was allowed to continue for 20 min, protected from light, and stopped by the addition of 50 μl 2 N H_2_SO_4_. The plate was immediately read at 450 nm. Duplicate standard and sample wells were averaged and background subtracted based on blank wells. TH concentration was calculated using a quadratic curve equation calculated in GraphPad Prism 8, and then normalized to total protein concentration per sample as calculated using Lowry assay. Final TH values shown are presented as pg TH/mg total protein after the multiplication of the nanogram TH value by 1000.

### HPLC

These experiments were performed via a blinded experimental design. Midbrain primary cultures were incubated in KH buffer (Table 1), at 37 °C, for 1 h before collecting intracellular and extracellular milieu for HPLC analysis^72,160^. For extracellular milieu, KH buffer incubated with neurons was collected, treated with 1 M perchloric acid, and snap frozen for analysis. For intracellular milieu, coverslips were washed with KH buffer, scraped, and treated with 1 M perchloric acid, before sonicating. Then, the sample was centrifuged at 12,000 r.p.m. at 4 °C for 10 min, and the supernatant was snap frozen in liquid nitrogen for analysis. The pellet was resuspended using 0.2 NaOH and RIPA buffer^161^ for protein quantification via Lowry assay. Samples were centrifuged at 16,000 × *g* for 15 min (4 °C) and the supernatant was filtered through a 0.2 mm pore membrane (Nanosep with 0.2 mm bioinsert, Pall Life Sciences) and 15 μl of the supernatant was injected directly into an HPLC-ECD (HTEC-510; Eicom). Dopamine was separated on a CAX column (EICOMPAK 2.0 i.d. × 200 mm) maintained at 35 °C. The mobile phase consisted of 70% 0.1 M ammonium acetate buffer (pH 6.0) containing sodium sulfate (0.025 M), EDTA-2Na (50 mg/l), and 30% methanol at a flow rate of 250 μl/min. An electrochemical detector that used a glassy working electrode (+450 mV) against a silver-silver chloride reference electrode (WE-3G; Eicom) was used to quantify dopamine in the samples. A dopamine standard was used to identify and quantify the dopamine concentration in the samples.

### Morphometric analysis

These experiments were performed via a blinded experimental design. Control (DAT^IRES*cre*^) neurons and α-syn-overexpressing neurons (DAT^IRES*cre*^/α-syn) were transduced with AAV1-LoxP-tdTomato (Addgene) on DIV5. Neurons were fixed with 4% PFA for 30 min at room temperature on DIV10, and coverslips were mounted using Fluoromount-G and allowed to dry. Alternatively, neurons were fixed, co-immunolabeled with TH and GFP antibodies, and mounted for imaging, as described above. Images were captured on a Nikon A1 laser-scanning confocal microscope (visualized through a ×20 oil-immersion objective). Images of neurons with minimal interference from neighboring neurons were analyzed in ImageJ (FIJI) and converted to 8-bit binary images after threshold adjustment. Sholl analysis plugin was used to draw concentric circles starting from 15 μm, followed by 5 μm successive shells in order to identify the number of intersections along the radii^108,162,163^. Sholl analysis was performed, and the number of intersections was plotted (Fig. 6). Cell area measurements were attained by manually drawing ROI around cell soma using the free polygon selection tool in ImageJ. ROIs were drawn to encompass the complete projection area of the cell and selection was finalized by a convex hull to attain final projection area measurement. Results obtained were plotted to analyze the complexity of morphology.

### Statistical analysis

Data analysis was performed using GraphPad Prism version 8.02 and MATLAB version 2020a. Student’s *t* test, linear regression, one-way, two-way, or repeated-measures ANOVA were used where appropriate and corrected for multiple comparisons. The significance of *P* < 0.05 was considered statistically significant. Data are presented with a mean and standard error, unless otherwise stated.

### Limitations to the methodology and model system used in this study

The primary neuronal culture used in this study is derived from the ventral midbrain, which contains the dopaminergic nuclei SNc and VTA. Notably, it has been shown that SNc dopaminergic neurons are more sensitive than VTA dopaminergic neurons^22,23,26–31,164^. Therefore, this model is likely to contain more VTA dopaminergic neurons than SNc dopaminergic neurons^22,26–31^. In addition, α-syn-mediated neuronal loss can also lead to a higher loss of SNc dopaminergic neurons than VTA dopamine neurons. Since functional analyses are conducted on surviving neurons, our data may overrepresent effects in the surviving VTA midbrain dopamine neurons.

In this study, we utilized TH promoter-driven expression of GFP and α-syn to confirm the specificity of TH promoter-dependent viral expression. We also performed calcium imaging, TH expression, and neuronal cell count in dopaminergic neurons expressing control vector (AAV-TH-GFP). We found that, compared to naive neurons, the AAV-TH-GFP transduction did not change neuronal activity and calcium dynamics in response to dopamine and quinpirole exposure, TH levels, and cell count (data not shown); therefore, the control AAV was not used for the rest of the experiments.

In regards to investigating if α-syn overexpression in dopaminergic neurons alters D2R expression, we utilized the biotinylation assay to assess the differences in membrane vs. cytoplasmic D2 levels^91–94^. Whether or not the detected membrane D2Rs are functional or desensitized remains unclear.

### Reporting summary

Further information on research design is available in the Nature Research Reporting Summary linked to this article.

## Supplementary information

Supplementary Information

Reporting Summary

## Data Availability

The data acquired and analyzed for this study are available from the corresponding authors upon reasonable request.
