# Supplementary material for: α-Synuclein-induced dysregulation of neuronal activity contributes to murine dopamine neuron vulnerability
Source: NPJ Parkinsons Dis. 2021 Aug 18;7:76. doi: 10.1038/s41531-021-00210-w (PMC8373893; doi:10.1038/s41531-021-00210-w)
Supplement: Supplementary file 1 — Supplementary Information [file 41531_2021_210_MOESM1_ESM.pdf]

Supplemental Figure 1

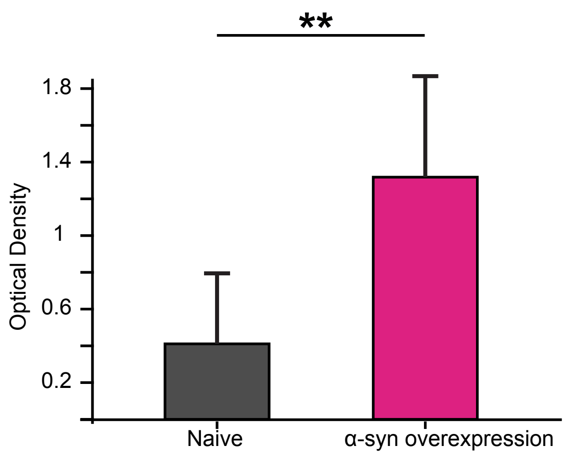

Supplemental Figure 1: AAV transduction of TH-dependent α-synuclein produces robust overexpression. Quantification of western blots illustrating increased α-syn levels in naive and transduced cultures (n=4, two-tailed t-test, p=0.005).

Supplemental Figure 2

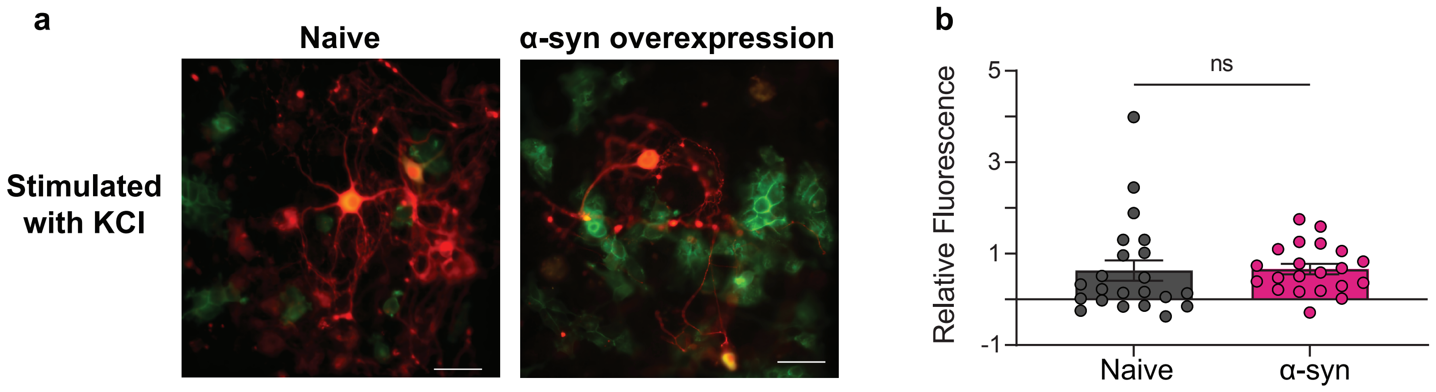

Supplemental Figure 2: KCl stimulated dopamine release is detected by GRABDA2M dopamine sensor. (A) GRABDA2M cells co-cultured with naive (left) or α-syn overexpressing neurons (right) following stimulation with KCl (90mM) to induce dopamine release. Upon stimulation, (B) naive and alpha-synuclein overexpressing dopamine neurons release similar amounts of dopamine. To compare KCl-stimulated dopamine release, the changes on the average fluorescence signal of cells adjacent to the neuron soma and neuronal processes before and after KCl were calculated (Relative Fluorescence = (Fstimulated – Fbaseline) /Fbaseline). These experiments confirm that the increased in GRABDA2M fluorescence signal is indeed due to dopamine release.

Supplemental Figure 3

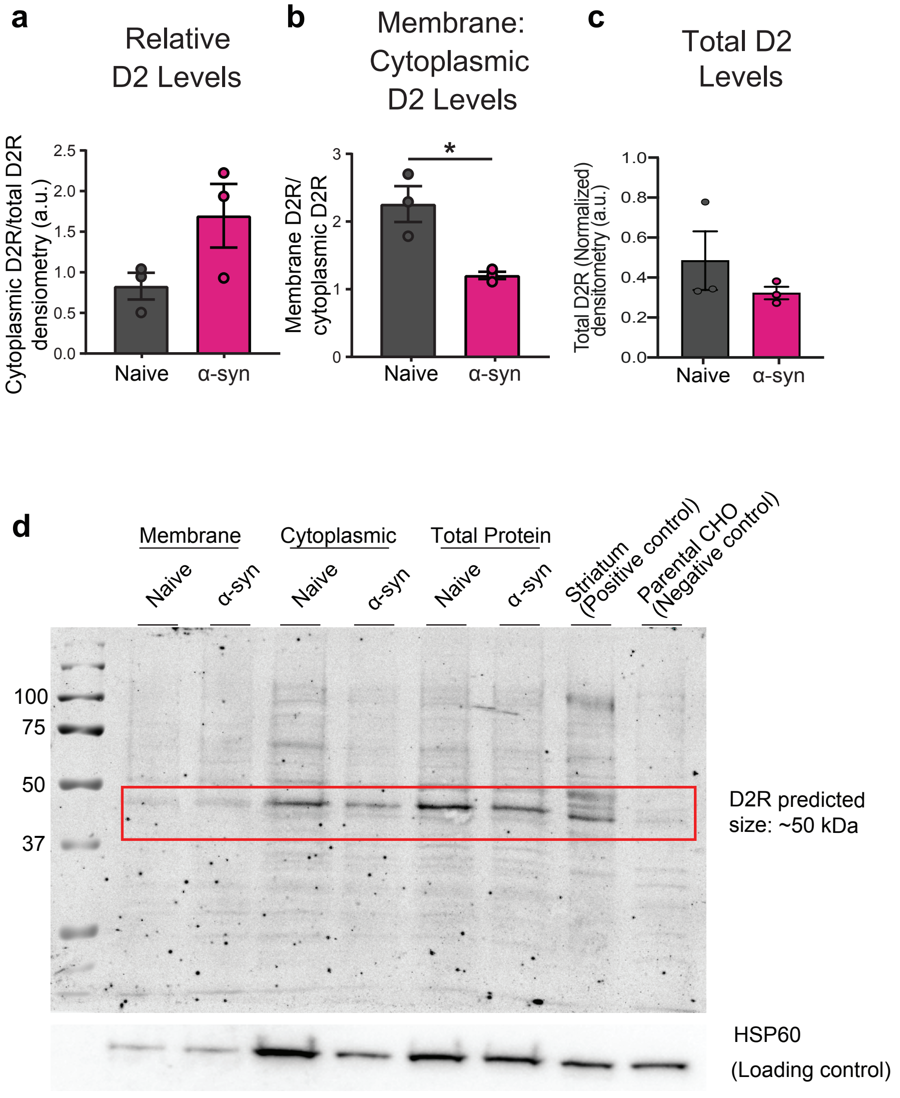

Supplemental Figure 3: Western blot analysis of D2 expression to Figure 5. (A-C) Membrane biotinylation experiments. Membrane and cytoplasmic D2 factions were isolated from a separate set of naive and α-syn overexpressing neurons, prepared identically, via a blinded experimental design. (A) α-syn overexpressing neurons exhibit unaltered levels of cytoplasmic D2 receptor relative to total D2 receptor levels (n=3 from independent replicates, two-tailed unpaired t-test p=0.1115). (B) The ratio of average membrane D2R to the average cytoplasmic D2R significantly decreases in α-syn overexpressing neurons (n=3 from independent replicates, two-tailed unpaired t-test p=0.0178). (C) Total D2R normalized to loading control HSP60 shows no significant difference in total D2R levels. The data are presented as mean ± SEM with independent replicate data points overlaid. (D) Representative blot for D2R shown. Mouse striatum lysate was used as the positive and parental CHO cells as negative controls for D2R expression. HSP60 was used to confirm membrane and cytoplasmic fractions.
